# Epithelial organ shape is generated by patterned actomyosin contractility and maintained by the extracellular matrix

**DOI:** 10.1371/journal.pcbi.1008105

**Published:** 2020-08-20

**Authors:** Ali Nematbakhsh, Megan Levis, Nilay Kumar, Weitao Chen, Jeremiah J. Zartman, Mark Alber

**Affiliations:** 1 Department of Mathematics, University of California, Riverside, Riverside, California, United States of America; 2 Interdisciplinary Center for Quantitative Modeling in Biology, University of California, Riverside, Riverside, California, United States of America; 3 Department of Chemical and Biomolecular Engineering, University of Notre Dame, Notre Dame, Indiana, United States of America; 4 Bioengineering Graduate Program, University of Notre Dame, Notre Dame, Indiana, United States of America; 5 School of Medicine, University of California, Riverside, Riverside, California, United States of America; 6 Department of Bioengineering, University of California, Riverside, Riverside, California, United States of America; Oxford, UNITED KINGDOM

## Abstract

Epithelial sheets define organ architecture during development. Here, we employed an iterative multiscale computational modeling and quantitative experimental approach to decouple direct and indirect effects of actomyosin-generated forces, nuclear positioning, extracellular matrix, and cell-cell adhesion in shaping *Drosophila* wing imaginal discs. Basally generated actomyosin forces generate epithelial bending of the wing disc pouch. Surprisingly, acute pharmacological inhibition of ROCK-driven actomyosin contractility does not impact the maintenance of tissue height or curved shape. Computational simulations show that ECM tautness provides only a minor contribution to modulating tissue shape. Instead, passive ECM pre-strain serves to maintain the shape independent from actomyosin contractility. These results provide general insight into how the subcellular forces are generated and maintained within individual cells to induce tissue curvature. Thus, the results suggest an important design principle of separable contributions from ECM prestrain and actomyosin tension during epithelial organogenesis and homeostasis.

## Author summary

The regulation and maintenance of an organ’s shape is a major outstanding problem in developmental biology. An iterative approach combining multiscale computational modelling and quantitative experimental approach was used to decouple direct and indirect roles of subcellular mechanical forces, nuclear positioning, and extracellular matrix in shaping the major axis of the wing pouch during the larval stage in fruit flies, which serves as a prototypical system for investigating epithelial morphogenesis. The research findings in this paper demonstrate that subcellular mechanical forces can effectively generate the curved tissue profile, while extracellular matrix is necessary for preserving the bent shape even in the absence of subcellular mechanical forces once the shape is generated. The developed integrated multiscale modelling environment can be readily extended to generate and test hypothesized novel mechanisms of developmental dynamics of other systems, including organoids that consist of several cellular and extracellular matrix layers.

## Introduction

Epithelial tissues are critical drivers of morphogenesis [1–3]. Functionally, they serve as barriers between the environment and internal structures of organs. Bending and folding are common features of many epithelial tissues [4]. However, a predictive understanding of how organs regulate their shape at a given stage of the development remains elusive. This is partially because the roles of mechanical properties of components of cells and tissues during organ development are hard to quantify experimentally. Further, the interactions between subcellular components that define tissue level-properties are non-linear, non-intuitive, and time-varying. Elucidating general design principles that can explain the overall mechanisms governing epithelial morphogenesis remains a key goal for characterizing multicellular systems [5–7]. Consequently, computational modeling approaches coupled to experimental studies are becoming powerful new tools to infer and test the basic design principles of epithelial morphogenesis.

**Fig 1:**
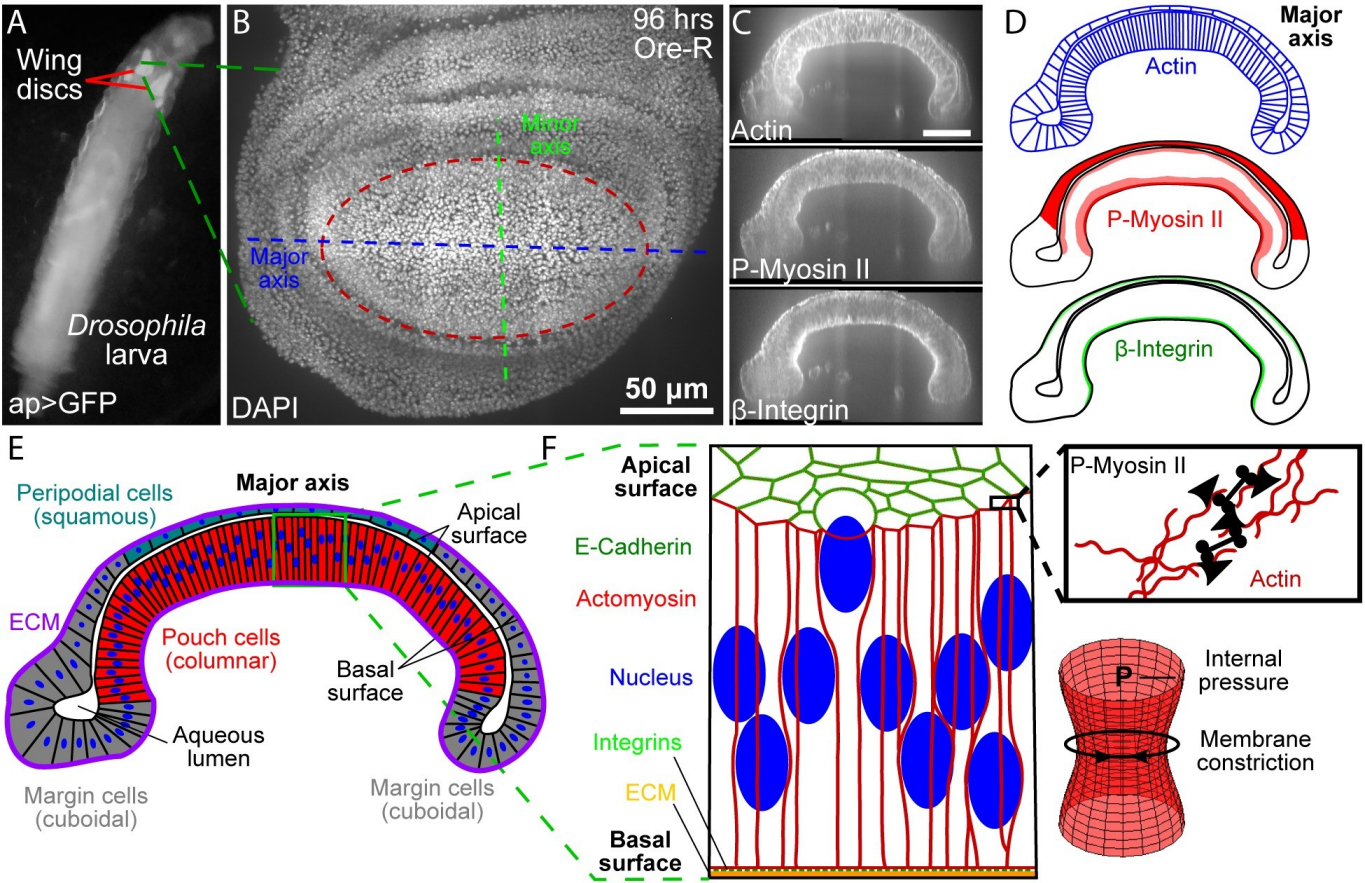
Cross-section of wing imaginal disc along the anterior-posterior axis. (A) Imaginal wings discs in 3^rd^ instar *ap>GFP* larvae. (B) Top view of a wing disc at 96 hours after wing disc in B stained for actin (phalloidin), P-Myosin II, and β-Integrin. (C, D) Patterns of Actin, P-Myosin II, and β-Integrin in the cross-section that follows the major axis of the wing disc pouch. (E) Multiple cell types within the wing disc. The wing disc is composed of squamous peripodial cells (top, blue), columnar pouch cells (bottom center, red), and cuboidal marginal cells (sides, grey). An aqueous lumen is enclosed by the apical surface of the two cell layers. The basal surface is constrained by the extracellular matrix (ECM). (F) Structural components of the *Drosophila* wing disc columnar cells (left). Schematic showing for actomyosin contractility (right, top). An actin mesh provides structural support to the cell membrane. Actin filaments are pulled together by phosphorylated non-muscle Myosin II (P-Myosin II) resulting in increased membrane tension. Actomyosin driven tension opposes the internal pressure in cells and acts to constrict the membrane (right, bottom).

The *Drosophila* (fruit fly) wing imaginal disc serves as a paradigm system to study epithelial morphogenesis (Fig 1) [8–10]. A genetic and biophysical toolkit that includes recent advances in organ culture and live-imaging techniques has been developed to investigate mechanisms underlying the shape formation of a wing disc [6,7]. During larval stages (1^st^, 2^nd^, and 3^rd^ instar), the wing disc undergoes a period of rapid growth with significant shape changes from a round epithelial vesicle consisting of a single epithelial monolayer [10,11]. At early stages of development, the wing disc, consisting of cuboidal cells, develops into a stereotypically folded tissue with multiple classes of epithelial cells, including squamous, cuboidal and pseudostratified columnar cells [12]. In mid- to late larval stages, the wing pouch forms multiple folds along the dorsal-ventral axis while a characteristic bent “dome” shape in the cross-sectional profile along the anterior-posterior axis is stably maintained (Fig 1C–1E, Fig A in S1 Text) [13–15]. The stereotypical shape of the wing disc plays an important role defining the initial geometric condition leading to pupal morphogenesis. During pupal morphogenesis, the wing disc undergoes a series of morphogenetic steps to form the adult fly wing [10,16,17]. A failure to achieve and maintain this stereotypical folded and curved shape at the end of larval development distorts subsequent stages of wing disc morphogenesis, resulting in misshapen wings. In turn, the final wing shape is critical for ensuring adequate flight performance [18].

Relative concentrations of key cytoskeletal structural, motor and adhesion proteins including actin, phosphorylated non-muscle Myosin II (P-Myosin II), and β-integrin are patterned within the larval wing disc (Fig 1C and 1D). The cross-section of a 96 h after egg laying (AEL) wing disc consists of squamous (peripodial) and columnar (pouch) cell layers that adhere to each other along their apical surfaces. Connected together, these cells enclose an aqueous lumen [19]. A thin extracellular matrix (ECM) forms a basement membrane that wraps around the cells along their basal surface (Fig 1E and 1F). Integrins adhere the ECM to the basal surface of cells while the integrity of the apical surface is maintained by intercellular adhesion junctions (Fig 1F). An actin mesh containing phosphorylated (P-Myosin II) termed the actomyosin generates internal stresses and provides structural integrity to individual cell membranes (Fig 1F). Actomyosin promotes membrane constriction and thus opposes internal cellular pressure.

Folding mechanisms have been widely explored for multiple tissues, including the developing wing disc [20–31]. However, how subcellular processes contribute to the global shape of the tissue is a fundamental, unanswered question. In this study, we investigated the contributions of multiple cellular processes to the regulation of the overall stereotypical dome shape of the wing disc pouch along the major anterior-posterior (AP) axis. To do so, we combined quantitative experimental analysis with a newly developed and biologically calibrated, subcellular element-based computational model. In the computational model, we included cell membrane, nuclear shape and position, and homotypic and heterotypic cell-cell and cell-ECM interactions. In the experimental analysis, we measured multiple features of wing discs at 96 h AEL, including nuclear positioning, cell height, and tissue shape in the wild type condition. We also imaged the spatial distribution of actomyosin, collagen, and integrins. These experimental data provided important inputs to calibrate the computational model.

Results from computational model simulations and quantitative analysis of genetic and pharmacological perturbations of the wing disc were integrated to explain how subcellular dynamics impact the formation of the tissue level features of the wing. We found that patterned actomyosin contractility explains the global bending shape generation along the AP axis observed in experiments. Surprisingly, actomyosin contractility is not required for maintaining the bending shape. While patterned pre-straining of the underlying ECM only played a minor role in generating the curved tissue shape along the AP axis of the organ, it was found to be critical for maintaining the curved dome shape once generated. Lastly, perturbation studies demonstrated that maintaining regional apical adhesion between the two cell layers within the wing disc requires ROCK-driven actomyosin activity in the absence of the basal extracellular matrix.

## Results

### The distribution of actin, phosphorylated myosin II and *β*-integrin is patterned along the wing disc cross-section

**Fig 2:**
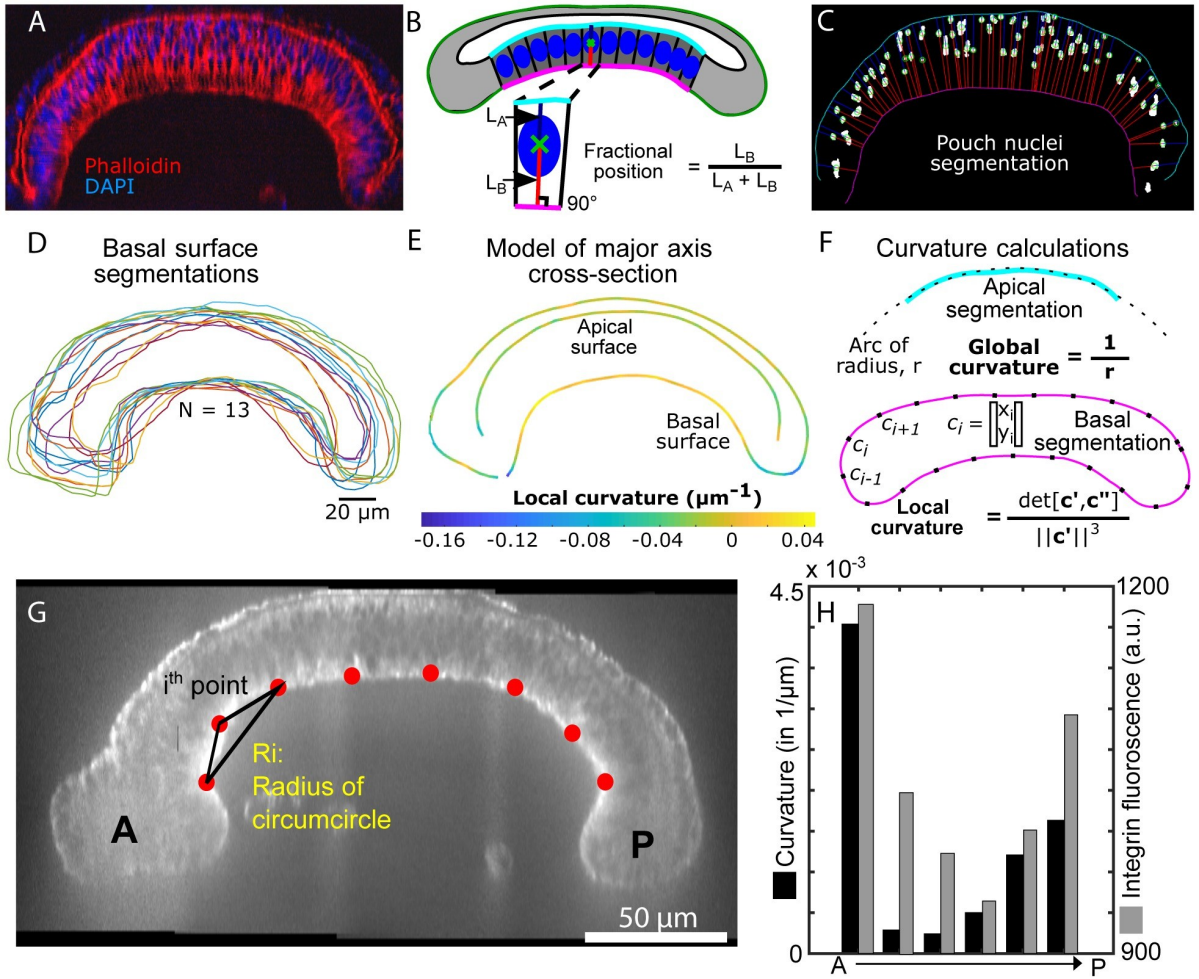
Quantitative analysis of wing disc shapes and nuclear positions along the major axis of imaginal wing discs at 96 hours AEL. (A) Representative pre-processed image of a wing disc cross-section stained with DAPI (nuclei) and phalloidin (staining F-actin). These images were used segment nuclei and surfaces defining the wing disc shape. B) The fractional position between the apical and basal surface serves as a metric for quantifying relative nuclear positions. Note: heights of columnar pouch cells were calculated as the sum of L_A_ and L_B_. C) Representative segmentation of nuclei. Blue and red lines correspond to L_A_ and L_B_ in B, respectively. D) Basal surface segmentation for multiple samples in different colors (N = 13). E) Composite representation of wing disc morphology generated from samples in D. F) Quantification of global and local curvatures. Fig A and Fig B in S4 Text provide additional details on the image processing pipeline. G) Representative image highlighting average curvature quantification used for H, the curvature was found using a circumcircle approach. H) Curvature quantification of regions of interest along the pouch as well as intensity quantification of integrin along the basal side of the pouch.

Quantitative spatiotemporal maps of subcellular components are required to develop integrative models of organogenesis. We first quantitatively mapped the primary structural components involved in regulating the mechanical properties of cells in 3^rd^ instar wing discs. Discs were obtained at multiple stages and stained for key architectural components of cells (Figs 1 and 2, and Fig A in S1 Text). To our knowledge, the biophysical mechanisms determining the overall cross-sectional profile along the AP axis have not been quantitatively investigated. Visualization of fluorescent intensities along the major axis in this cross-section showed that filamentous actin (F-actin) was consistently localized to cell membranes (Fig 1C and 1D).

Phosphorylated non-muscle Myosin II (P-Myosin II) was seen in squamous cells and at higher intensity levels in columnar pouch cells within a narrow strip along the apical surface and a wider strip between the basal surface and nuclei (Fig A in S1 Text and Fig D in S4 Text). β-integrin was concentrated basally in columnar cells and to a lesser degree in squamous cells (Fig 1C and 1D, Fig A in S1 Text and Fig D in S4 Text). These protein distributions suggest actomyosin-driven activity is significant in squamous cells and the apical surface and basal compartment beneath cell nuclei in columnar cells. Observed cell shapes suggest that this contractility occurs laterally in squamous cells resulting in flatter cells and apicobasally in columnar pouch cells, such that pouch cells become narrower and more elongated. The differences in β-integrin intensity associated with squamous and columnar cells suggest differential relative integrin-ECM adhesion between the two layers and possible heterogeneity in passive tension levels in the underlying extracellular matrix, which requires additional analysis.

Additionally, fluorescently stained nuclei exhibit apically-biased positions in columnar pouch cells (Fig 2A). We segmented nuclei and the apical and basal surfaces of the pouch region to quantify the fractional apicobasal nuclear position (Fig 2B and 2C). This revealed that nuclear positions in columnar cells are biased toward the apical surface (Fig A and Fig B in S4 Text). This nuclear migration in wing discs depends on actomyosin contractility [32]. Since the actomyosin is present at the basal side of the columnar cells, we hypothesized that actomyosin may also contribute to the tissue-level dome, or bent, shape of wing disc along the AP axis.

### The curvature of the basal surface correlates with local β-integrin concentration levels

Images of wing discs stained for actin and nuclei were processed to extract apical and basal surface segmentations (Fig 2D). The extracted profile measured by the segmentation framework (Fig A, Fig B and Fig C in S4 Text) provides generalized shape information of the wing disc cross-section, including tissue thickness and local curvature measured for the composite pattern of the basal surface for experimentally captured tissues (Fig 2E). A comparison of this local curvature profile to the distribution of β-integrin (Fig 2G and 2H) reveals a correlation between basal curvature and β-integrin intensity. Marginal cells at the lateral edge of the tissue (here termed boundary cells) had a reduced signal of β-integrin accumulation and exhibited a strongly negative basal surface curvature. The basal surface associated with squamous cells had a higher amount of β-integrin localization than boundary cells and slightly negative basal curvature. In the pouch region, β-integrin density was higher than both boundary cells and squamous cells, and the corresponding basal curvature was positive.

**Fig 3:**
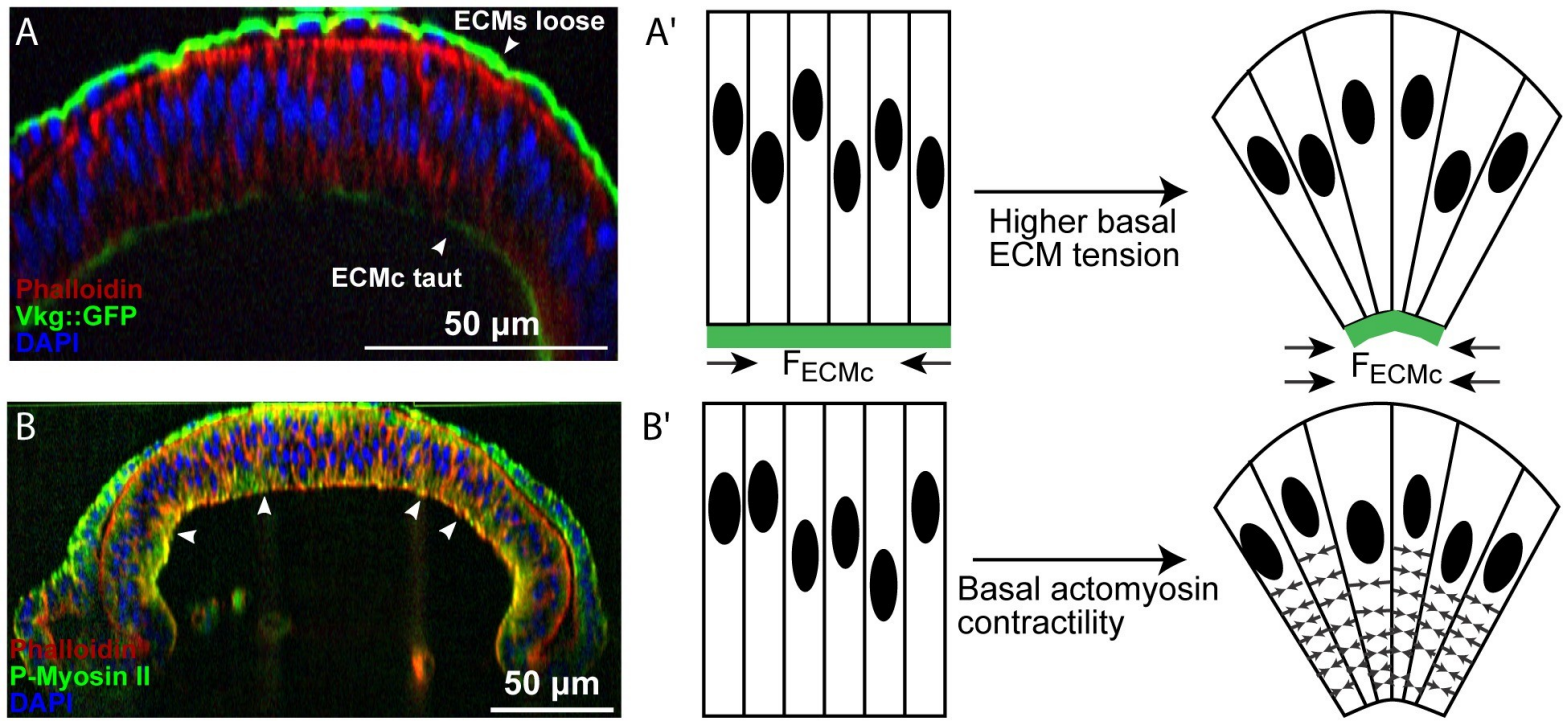
Potential mechanisms of wing disc bending. (A) The patterned ECM tension hypothesis. In this hypothesis, the level of passive tension of the ECM is higher next to the columnar cells compared with ECM next to squamous cells. (A’) Graphical illustration of potential hypothesis explaining how high ECM tension at the basal side of the columnar cells compresses the tissue and contributes to curvature profile of wing disc. (B) The patterned actomyosin contractility hypothesis. The columnar cells were stained for actin by Phalloidin and an antibody to P-Myosin II. (B’) Schematic of hypothesized mechanism for generating epithelial bending and asymmetrical nuclear positioning in the wing imaginal disc. Actomyosin contractility beneath the nucleus of columnar cells drives wing disc bending.

These observations suggest that β-integrin-associated adhesion results from an accumulation of passive ECM tension. Imaging of wing discs with fluorescently labeled Collagen IV (Viking::GFP) revealed that the ECM was relatively loose around the peripodium. In contrast, it is taut along the basal side of the pouch (Fig 3A), further supporting a possible role for β-integrin-mediated regulation of ECM tension.

### Experimental observations require a multiscale mechanical model to determine mechanisms of relative contributions of subcellular components to overall tissue shape

The apically biased nuclear positioning in the pouch region suggests a possible mechanism for bending the wing disc along the major anterior-posterior axis of the pouch (Fig B in S4 Text). Columnar cells are wider near the apical surface, which is consistent with nuclei being an order of magnitude stiffer than cytoplasm[33]. This would, in turn, tend to result in stretching and bending forces on the squamous cells that consist define the top layer of the wing disc. Further, the apical asymmetry in the nuclear position possibly facilitates bending of the tissue (Fig 2). Thus, we hypothesized that actomyosin-driven forces might play a dominant role in maintaining all nuclei near the apical side in columnar cells, also resulting in the bending shape of the epithelial monolayer (Fig 3B–3B’). On the other hand, experiments also suggest that the ECM provides lateral compression to the wing disc (Fig 3A–3A') as the columnar pouch cells flatten out and become more cuboidal upon chemical digestion of the ECM with collagenase [34,35]. However, whether passive ECM tensile forces or active actomyosin contractility can individually account for either generating or maintaining the bent shape along the anterior-posterior axis remains unclear [15].

Disentangling the interplay between subcellular architectural components including actomyosin, integrin, and extracellular matrix and their associated mechanical forces is experimentally challenging. Thus, we created a combined computational model and experimental approach described in the Materials and Methods section to test proposed mechanisms individually. We formulated a subcellular element model that enables us to simulate the subcellular components of cell architecture in detail (Computational model subsection in Materials and Methods section). A detailed two-dimensional multi-scale model reasonably describes the essential geometric features of the wing disc pouch. In particular, the curved shape along the major anterior-posterior axis (Fig 1B, Fig A in S1 Text) is representative of the characteristic “dome” shape throughout the pouch region.

We incorporated into the computational model mechanical spring-like forces generated by actomyosin at the subcellular level, passive tension within the ECM, and adhesion between two cell layers consistent with previous observations [19,36,37] to investigate their individual functional roles. Composite experimental profiles used throughout this study provided both qualitative and quantitative comparisons with predictions from computational simulations.

### A high level of passive ECM tension next to columnar cells is required to bend the tissue

Recent work has demonstrated that the ECM actively contributed to the morphology of epithelial cells in the pouch [34,38]. Removal of the ECM reduced lateral compressive forces on the columnar cells in the *Drosophila* wing disc and led to shorter and fatter cells (Fig C in S4 Text). Therefore, these previous results suggest that the ECM is under tensile stress and it in turn compresses the cells. Consequently, we investigated whether the passive tensile stresses within the ECM was necessary and sufficient to generate epithelial bending of the wing disc.

As previously mentioned, the imaginal wing disc is enclosed by the ECM, which connects to the basal side of individual cells through integrin-mediated adhesion [38]. ECM remodeling follows the growth and division of epithelial cells in the wing disc [12]. A high growth rate of epithelial cells in comparison with ECM remodeling can lead to the accumulation of tensile stress in the ECM [39]. In other contexts, cell stresses are converted into pre-strains within the ECM [40]. Cell division happens more frequently in the columnar pouch cells than in the squamous peripodial cells [12]. This led us to assume that the tensile stress is higher in the basement membrane associated with the columnar cells. Experimental images confirmed that assumption as the ECM is relatively loose around the peripodium whereas it appeared taut around the pouch (Fig 3A and 3B).

**Fig 4:**
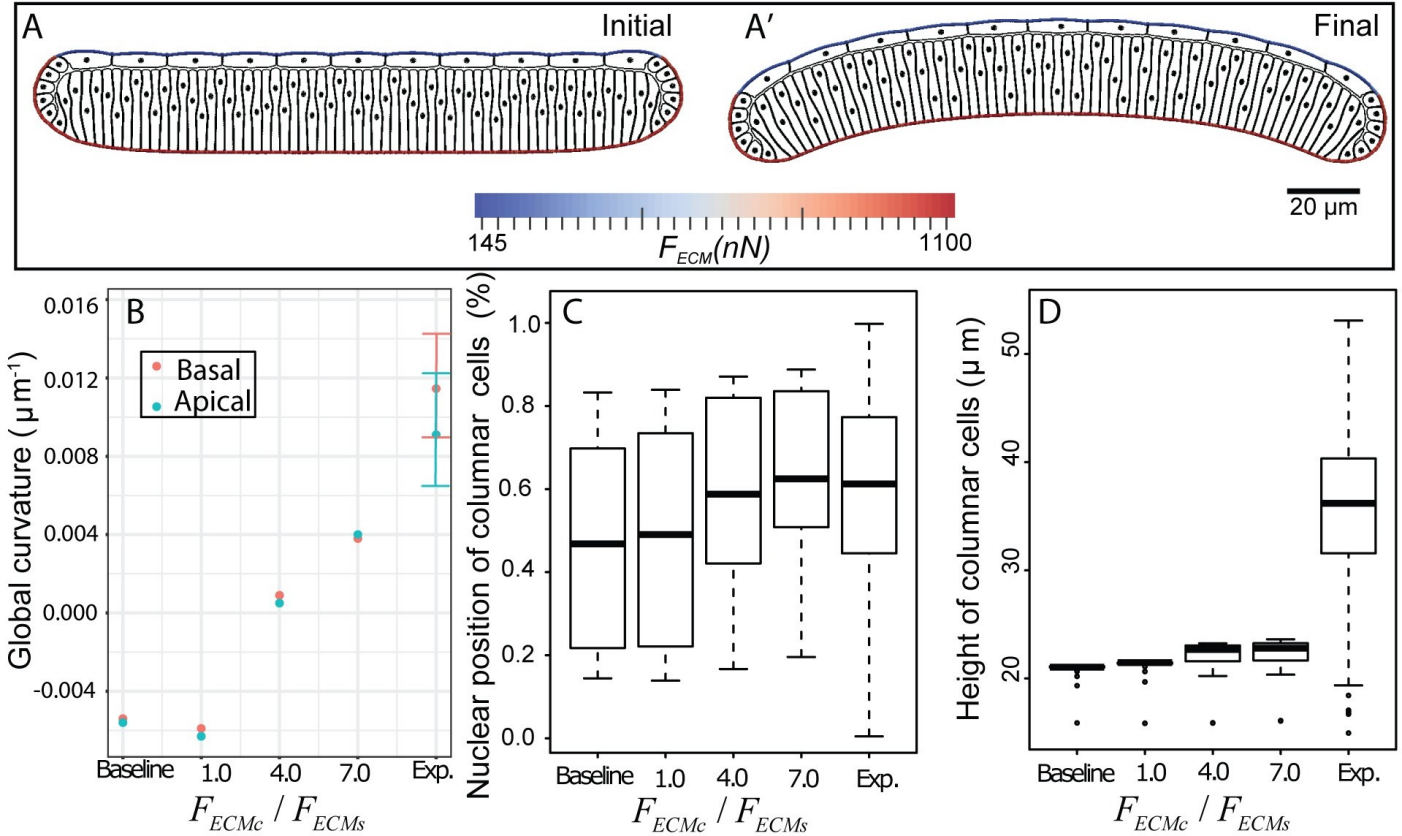
Computationally testing the hypothesis of patterned ECM tension. In these simulations, the passive tensile stress of the ECM associated with squamous cells (*F*_*ECMs*_) is lower than the passive tensile stress of the ECM associated with columnar cells (*F*_*ECMc*_). Extremely different tensile forces, *F*_*ECMs*_ v.s. *F*_*ECMc*_, are needed to bend the tissue, (A) Initial and final frames of a representative computational simulation showing how higher tension in ECM associated with columnar cells in comparison with the tension in the ECM associated with squamous cells leads to slightly curved shape profile of the wing disc. Comparison of in-silico prediction of impacts of different levels of ECM tension on (B) curvature profile of experimental (n = 16) and simulated (n = 1) wing discs. Experimental data are shown with mean ± standard deviation. The final global curvature of the tissue is uniquely determined in the simulation with a specific set of input parameters; (C) the relative position of nuclei, (D) the height of columnar cells. In C and D, boxplots show minimum, first quartile, mean, third quartile, maximum, and outlier of simulated (n = 65) and experimental (n = 1064) columnar cells. “Baseline,” here, corresponds to the condition that the whole ECM is initiated without any tension in the simulation.

Additionally, there is significantly higher observed integrin intensity connecting the basal side of cells to ECMc (Fig 1C). We first tested the hypothesis that higher tensile stress in ECMc in comparison with ECMs can lead to the overall bending shape in the *Drosophila* wing disc (Fig 3A’). To test this, we performed computational model simulations without accumulated tension in the ECM (Fig 4B–4D, Baseline) as a baseline reference case to determine the shape of the tissue without a mechanism of active force generation. This baseline case was then compared to simulations with increased levels of tensile stress accumulated in the ECMc. Then we tested a model scenario with uniform tensile stress in both the ECMc and ECMs. Finally, we tested cases with differential stresses between each side of the tissue. As absolute levels were unknown, we varied the ECMc tensile stress from 4 to 7-fold higher than the tensile stress of the ECMs. All simulations start with a flat tissue and asymmetric nuclear distribution (Fig 4A) as the initial condition of the tissue morphology. Simulations are run to reach 50,000 in time with arbitrary unit (AU) using a time step size equal to 0.002 AU (Fig 4A’).

A comparison of simulated and experimental results in Fig 4B demonstrates that curvature profiles of the top and bottom surfaces of the simulated wing disc in all the cases are less than the curvature profiles obtained in corresponding experiments. Although differential tensile forces between ECMc and ECMs can bend the tissue globally, it requires an extremely large difference to reach the desired curvature profile of a wing disc as observed in experiments. None of the simulated discs bent as significantly as experimentally observed discs. Fig 4C shows that nuclear position follows similar distribution as the experimental data when the ratio *F*_*ECMc*_/*F*_*ECMs*_ becomes higher, i.e., there is a positive correlation between the ratio *F*_*ECMc*_/*F*_*ECMs*_ and nuclear position of columnar cells. No obvious correlation is observed between the ratio *F*_*ECMc*_/*F*_*ECMs*_ and the height of columnar cells. The overall results indicate that it is difficult to bend a wing disc tissue by the differential tensile forces between ECMc and ECMs alone. Only a mild bending phenotype can be obtained when much higher passive tensile stress is associated with ECMc than the stress associated with ECMs causing an asymmetric nuclear distribution.

### Basal actomyosin contractility is sufficient to induce tissue bending

Actomyosin plays an important role in shape formation during cell growth and tissue development [15,16,32]. For example, actomyosin contractility drives the nuclear motion in the columnar cells of *Drosophila* wing discs to enlarge the apical side during mitosis [32]. We hypothesized that actomyosin contraction beneath the nuclei of columnar cells not only maintains the asymmetrical distribution of nuclei along the apical-basal axis, but also bends the wing disc tissue (Fig 3B–3B’). Therefore, we performed a set of simulations with different levels of basal actomyosin contractility to test the global effect of basal actomyosin contractility beneath nuclei in columnar cells on tissue shape. To ensure we are only observing the effect of basal actomyosin contractility, we did not include any ECM tensile force at the initial time point.

**Fig 5:**
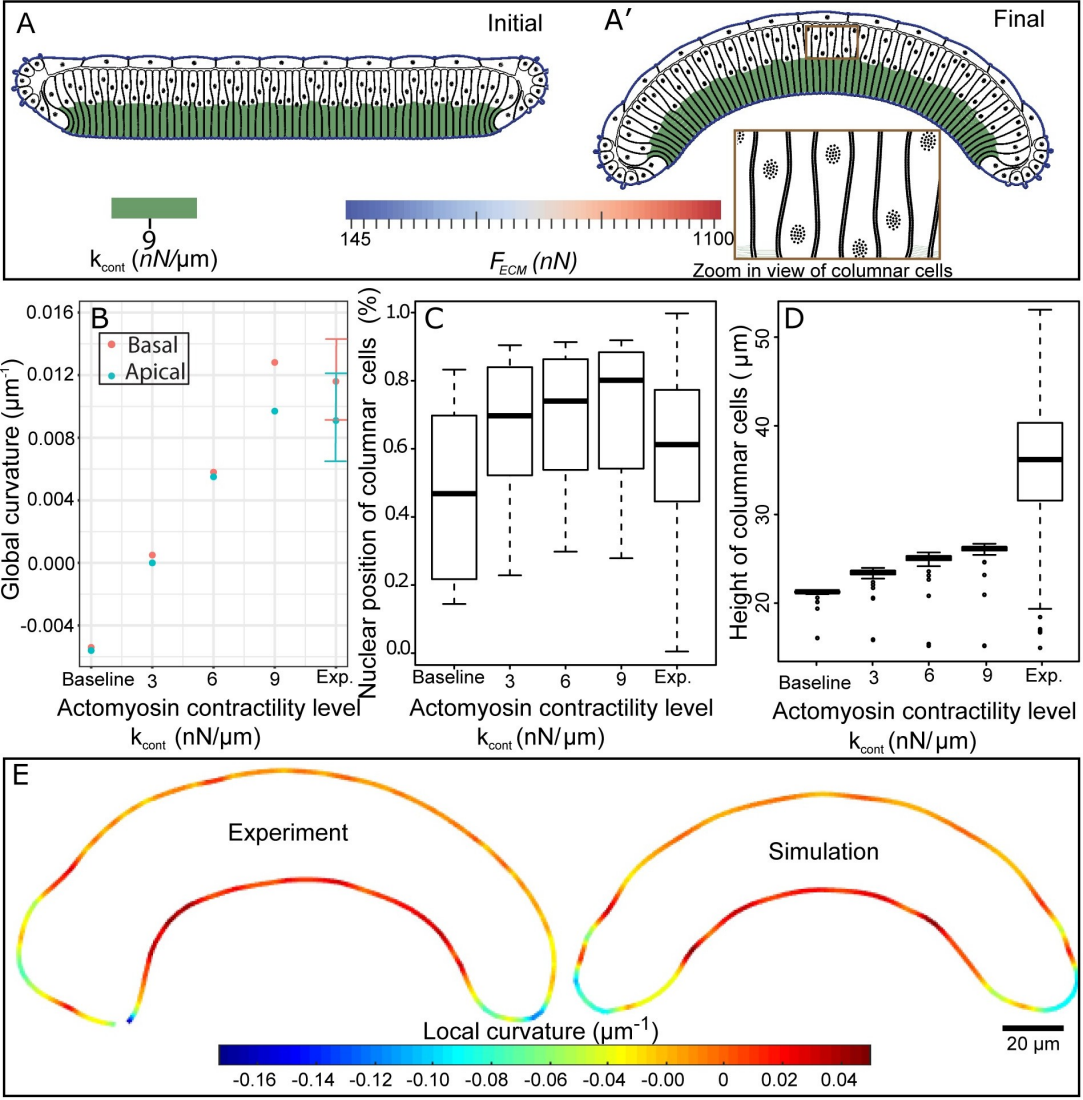
Quantitative and qualitative comparisons of experimental data with simulation results for different levels of simulated basal actomyosin contractility. (A) Initial and final frames of simulation result where actomyosin contractility leads to curved profile shape. (B) Global curvature of apical pouch surface and basal peripodial surface for experimental (n = 16) and simulated (n = 1) wing discs. Experimental data are mean ± standard deviation. (C) Relative nuclei positions of pouch cells. Note, the relative nuclei position has a value of zero at the basal surface and one at the apical surface. (D) Height of pouch cells. In C and D, boxplots show minimum, first quantile, mean, third quartile, maximum and outlier of simulated (n = 65), and experimental (n = 1064) columnar cells. Measurements for columnar cell heights experimentally and computationally were taken as the summation of the length of straight lines from apical to centers of segmented nuclei centers and from segmented nuclei centers to the basal side of the cells. (E) Comparison of local curvature profiles and shapes obtained from experimental data and computational results.

These simulations started with a flat tissue and asymmetric nuclear distribution, while the basal sides of pouch cells contracted as shown in Fig 5A. The simulations led to nuclear motion, changes in cell size and shapes, and tissue bending until the simulation reaches 50,000 AU. Three sets of simulations were performed with low (3 *nN*/*μm*), medium (6 *nN*/*μm*), and high (9 *nN*/*μm*) levels of actomyosin contractility. Each condition was then compared with both experimental data and the baseline simulations where there is no basal actomyosin contractility.

Simulations with different levels of basal actomyosin contractility demonstrated a reduction in the width beneath the nuclei of the columnar cells, resulting in the overall wing disc bending in the same direction as observed in experiments (Fig 5A’). The tissue curvature increased as a function of the level of actomyosin contractility (Fig 5B). Moreover, simulations demonstrated that the apically-biased nuclear distribution was maintained in columnar cells (Fig 5C), which is in agreement with recently reported experiments [41]. Heights of columnar cells also increased with increased contractility levels (Fig 5D), although it is still less than experimental data. A plausible reason for a reduced height of cells in simulations compared with experimental data is due to the fact that the initial height of columnar cells in the simulation were lower than the range of experimental data for computational cost efficiency. The local curvature profile along obtained in model simulations with high contractility is in a very good agreement with the profile observed in experiments (Fig 5E).

Application of the sensitivity analysis method based on Latin Hypercube sampling and partial correlation coefficient method [42] (S6 Text) confirmed that the overall shape (curvature) is primarily generated by patterned actomyosin contractility, with higher contractility below nuclei. This computational result confirms our hypothesis that the basal actomyosin contractility plays a significant role in bending the tissue along the AP axis. Model simulations predict that the actomyosin generates 9 *nN*/*μm* of force to produce the experimentally observed bent shape. Therefore, the asymmetric spatial distribution of nuclei for columnar cells is due to associated actomyosin contractility beneath the nuclei. This contractility-induced asymmetric distribution leads to the experimentally observed curved shape for the entire tissue. We propose this as the possible mechanism for generating the curved shape of the *Drosophila* wing along the anterior-posterior axis.

**Fig 6:**
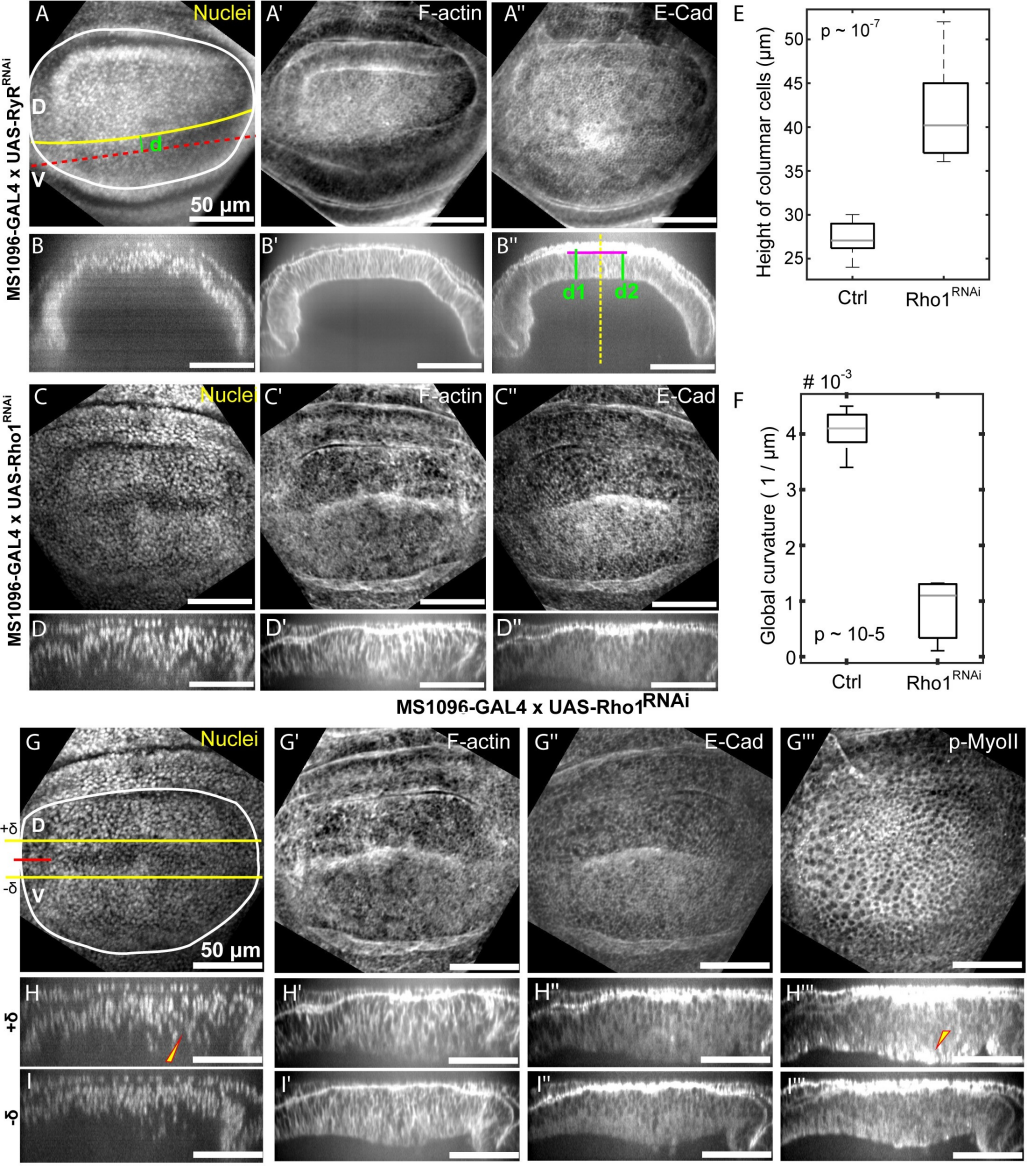
Rho1 promotes tissue bending and regulation of cell height. (A-B”) MS1096>RyR^RANi^ was used as a control (n = 5) for the comparisons. (A-A”) Representation of immunohistochemistry data using standard deviation z plane projections along with cross-sectional views along a line parallel to the AP axis. (C-D”) Loss of function of Rho1 in the wing imaginal disc was introduced using the Rho1^RNAi^ line (BL# 27727). (C-C”) Data corresponding to A-A”. (E) Rho1^RNAi^ (n = 8) in the wing imaginal discs leads to an increased columnar cell height as compared to control discs (n = 5). (F) Inhibition of Rho1 in the wing imaginal discs leads to flattening of the discs quantified through Menger curvature. A sample size of 5 and 8 was used for the control and Rho1^RNAi^ discs. (p-values of a student’s t-test included in plots) (G-I”) MS1096-Gal4 is expressed preferentially in the dorsal compartment of the wing imaginal disc. (G-H”‘) Representation of immunohistochemistry data using standard deviation z plane projections along with cross-sectional views along a line parallel to the D-V boundary. (H–I”‘) Cross-sectional views of the wing disc along lines parallel to the D-V boundary and located above and below the DV boundary as indicated by the yellow +δ and—δ lines in G.

To test the main prediction that active actomyosin contractility is essential for generating the curved profile of the wing disc along the anterior-posterior axis with cell height, we investigated the impact of knocking down Rho1, a key regulator of actomyosin contractility [43] (Fig 6). We used the GAL4/UAS binary expression system which allows for tissue-specific expression of RNAi constructs. The MS1096-Gal4 driver has higher expression in the dorsal compartment and limited expression in the ventral compartment. Based on previous research, we used an RNAi line that does not lead to qualitative phenotypic changes as a control (Ryr^RNAi^ expression by the same Gal4 driver). Columnar cell height was quantified by calculating average cell heights in the anterior and posterior side of the wing disc as shown in B”. Inhibition of Rho1 in wing imaginal discs led to a significant increase in columnar cell height (Fig 6E), in agreement with previous findings for inhibiting Rho1 [13]. Further, bending was quantified by calculating the local (Manger) curvature of the basal surface (Fig 6F). In agreement with computational model prediction, the inhibition of Rho1 in the wing imaginal discs resulted in a significant reduction in tissue curvature (p-value from t-test ~10^−5^).

We further investigated the impact on cell architecture of higher inhibition of Rho1 in the dorsal compartment using the ventral compartment as an internal control (Fig 6G–6I”’). Cross-sectional views of the wing disc were examined that were parallel to the AP axis and located in the dorsal compartment as indicated by the yellow lines (+δ or -δ). Interestingly, we found that knockdown of Rho1 resulted in more basally located nuclei and a reduction of F-actin in the basolateral regions of cells. Surprisingly, we also saw an increase in apically and basally localized phosphorylated myosin II (pMyo-II) as well as an increase in cell height. This could explain the decoupling of cell height and tissue curvature regulation. The implications for the deviations between experiments and simulations are explored in the discussion section.

### Tensile forces within the extracellular matrix maintain tissue bending

**Fig 7:**
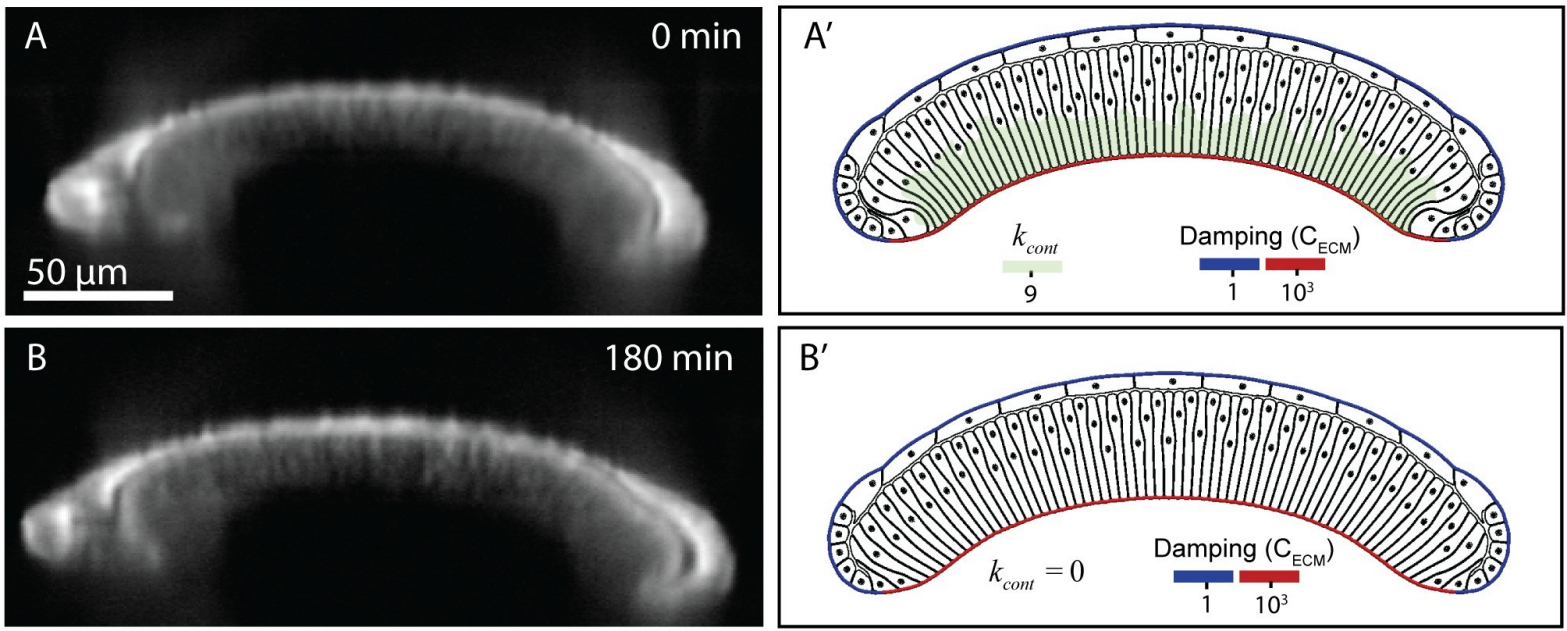
A comparison of experimental and simulated profiles demonstrates that ECM is sufficient to maintain the bent profile of the tissue. (A) Experimental profile before adding Latrunculin A to inhibit actomyosin. The wing disc was stained with CellMask. Note that the imaging conditions required for live-imaging do not provide as fine resolution as for fixed images. (A’) Computational model after the bent profile of the wing disc is formed with higher levels of basal actomyosin contractility. (B) Experimental profile three hours after the addition of 4 μM Latrunculin to inhibit actomyosin. (B’) Computational profile of wing disc showing that bend profile of wing disc is preserved even after removal of basal actomyosin contraction.

**Fig 8:**
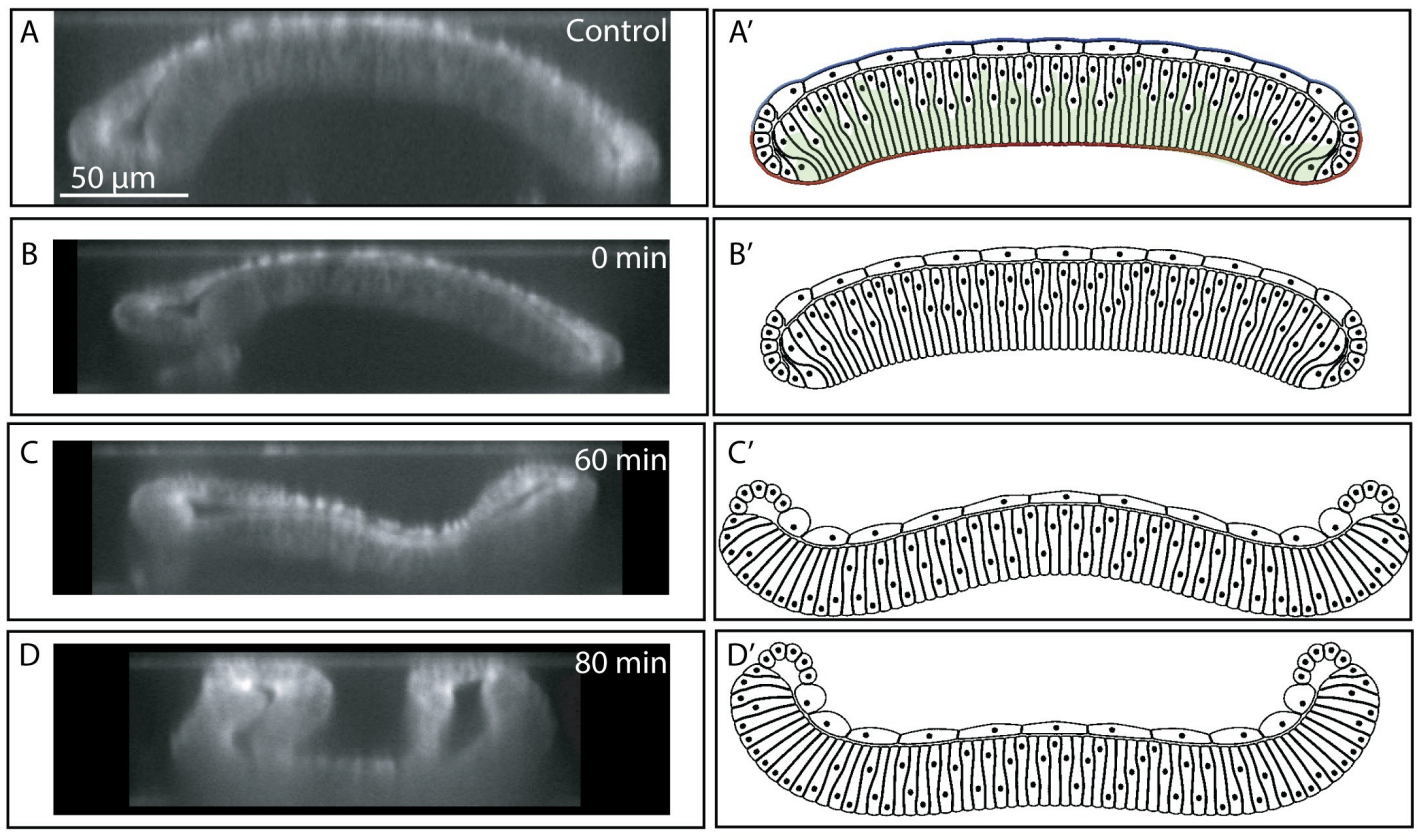
Enzymatic degradation of Collagen IV in 96 h wing discs. (A-D) Enzymatic degradation of the wing disc substantially changes the curvature profile of wing disc. (A’-D’) Computational simulation of acute removal of ECM and removal of actomyosin contractility components below the nuclear qualitatively match the experimental results. Wing disc was stained with CellMask for experimental imaging.

**Fig 9:**
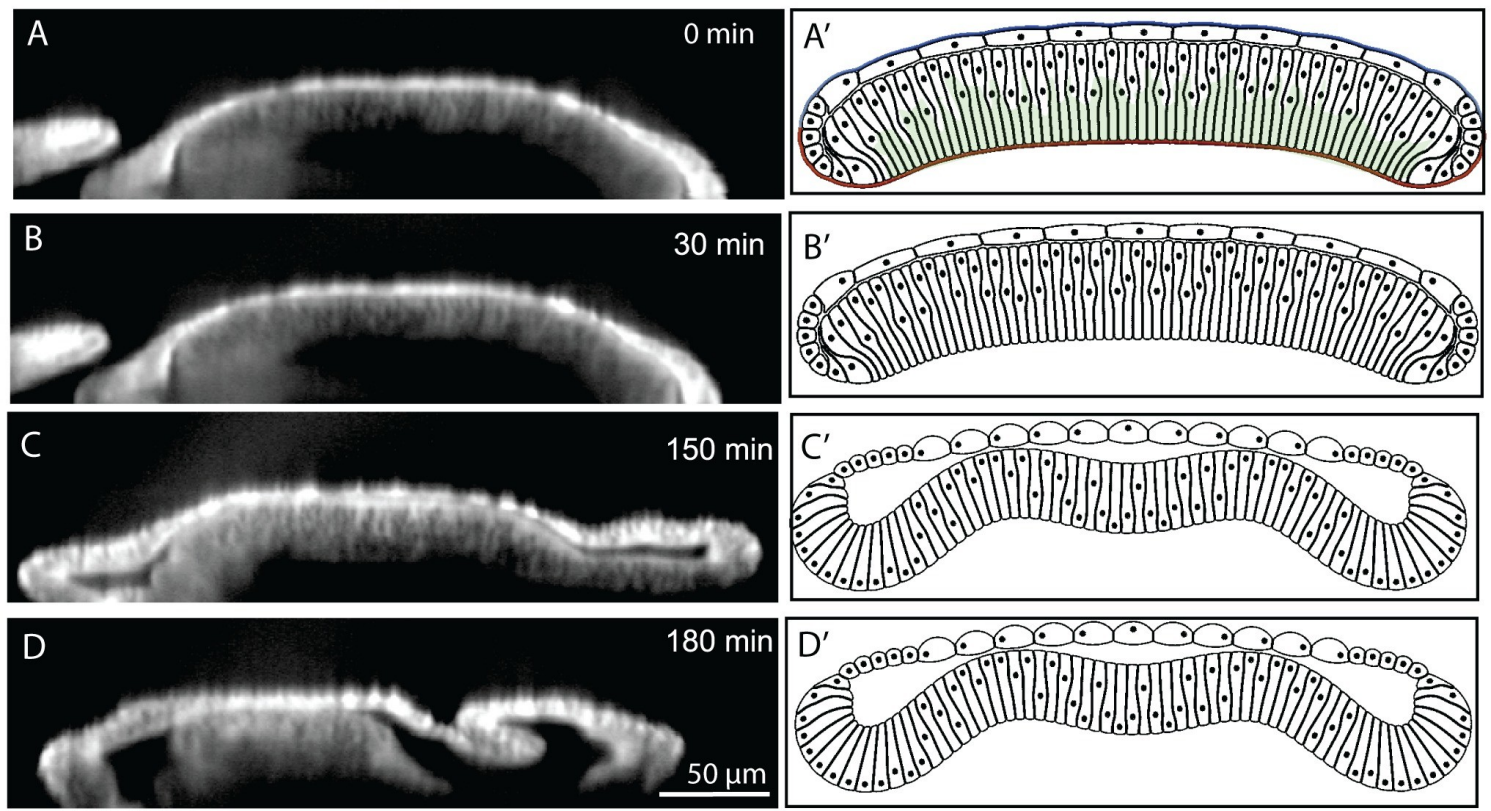
Enzymatic degradation of Collagen IV and actomyosin contractility in 96 h wing discs. (A-D) Enzymatic degradation of the wing disc in the presence of ROCK inhibitor to inhibit actomyosin contractility results in loss of adhesion between the squamous cells (top layer) and the central columnar cells. (A’-D’) Computational simulation of acute removal of ECM, removal of adhesion between cell layers, and removal of actomyosin contractility components below the nuclear qualitatively match the experimental results. The experimental wing disc is stained with CellMask.

In the previous sections, we tested whether the passive tension of the ECM or actomyosin contractility beneath the nuclei was sufficient to bend the *Drosophila* wing disc. To further understand the mechanism underlying the bending shape, we also studied the tissue shape under perturbation conditions in both experiments and simulations (Figs 7–9). Surprisingly, very little morphological change was observed when wing discs were incubated with Latrunculin A (Fig 7A and 7B) which is an inhibitor of actin polymerization. On face value, this result seemingly contradicts the inferred result of simulations presented in Fig 5B. We thus investigated whether higher resistance of the ECMc to the applied forces can explain this result based on short-term pharmacological perturbations.

Very recent experimental results support a significant role of the ECM in maintaining the shape of the tissue. Keller et al. demonstrated using finite element modeling and stretching experiments that the ECM is significantly stiffer than the columnar wing disc cells[44]. This provides additional evidence for compression by the ECM playing a defining role in maintaining the overall organ shape. In addition, Tozluoğlu et al. showed that ECM plays a major role in maintaining the folds in the wing disc in the orthogonal dorsal-ventral direction [39].

Therefore, to explain the experimental observations, we hypothesized that after the bent profile of wing disc is “generated” by basal actomyosin contractility, the ECM becomes resistant to deformation. Thus, we infer that ECM serves as a “memory” of tissue shape to preserve the bent shape of the tissue but does not generate the tissue shape. To represent this, we simulated the generation of the bent profile of wing disc. This was followed by computationally turning off the basal contraction in the simulation while increasing the damping coefficient of the columnar-associated ECM (ECMc) by three orders of magnitude (Fig 6A and 6B). This allowed the ECM to preserve its shape and hence preserve the shape of the wing disc.

In comparison, we experimentally tested whether inhibition of the actin polymerization with Latrunculin A would impact the tissue shape in ex vivo organ cultures [15]. Surprisingly, we found that inhibition of actin polymerization had a minimal impact on tissue shape maintenance (Fig 7A and 7B). This agrees with computational simulations indicating that a high mechanical resistance of the ECMc is sufficient to preserve the generated shape (Fig 7A’ and 7B’).

As further validation, we imaged the shape changes that occur when the ECM is chemically digested with collagenase in a 96 AEL wing disc. This perturbation resulted in the inversion of the bent wing disc pouch with the margin area curving toward the squamous cells (Fig 8C and 8D and Fig 8C’ and 8D’, S1 Video and S2 Video). This confirms that the ECM is essential for maintaining the overall curved cross-sectional profile along the AP axis. Thus, the ECM serves as a ‘morphogenic memory system’. Additionally, these experiments provide insight into a third mechanism that contributes to the mechanical stability of the wing disc and the formation of the final shape. Without the ECM, the pulling force from the squamous cells to the columnar cells transmitted through marginal cells bends the entire tissue in the opposite direction of the final bending shape. The same mechanism results in an inverted bending profile in the first two simulations in Fig 4B.

The squamous cells become thinner as larval development proceeds. Hence, the pulling force from squamous cells, working against the desired bending profile, could be decreasing to allow the wing disc to position itself in a curved profile to facilitate the next steps of pupal morphogenesis. Therefore, this combined perturbation study provides evidence that the ECM is contributing to the bent shape of tissue by mechanically preserving, but not generating, the generated shape of the tissue.

### Dual ECM digestion/ROCK inhibition reveals significant apical adhesion between columnar and squamous cells

We used a ROCK inhibitor (Y-27632) at 1 mM concentration to deactivate actomyosin contractility in addition to the removal of ECM with collagenase to further investigate the relative contributions of actomyosin and the passive prestrain of the ECM (Fig 9A–9D and S2 Video) [45,46]. Experimental results revealed that the tight connection between the apical side of columnar cells near the dorsal-ventral compartment boundary and squamous cells in the pouch region is lost after inhibiting the actomyosin contractility in conjunction with collagenase treatment. A clear gap was generated between the two layers at the center, similar to the genetic inhibition of Rho1 (Fig 6). Similar gaps were also observed in simulation results (Fig 9A’–9D’) when adhesion between columnar and squamous cells, is reduced to zero in combination to deactivating the parameter representing basal actomyosin contractility and ECM. This suggests that actomyosin contractility not only pushes the nucleus and cytoplasm toward the apical surface in the columnar cells, but it is also involved in supporting the localized adhesion between columnar cells and squamous. The adhesion between columnar and squamous cells are provided by microtubules extended between these two cell types[19]. Since microtubules are connected to the actin inside the cells, inhibition of ROCK may loosen the actin-microtubule network connection[47]. Hence columnar and squamous cells are not connected anymore, and a gap forms apically between the two layers. To summarize, this dual perturbation reveals a hidden mechanical role of the adhesion between the two layers of cells in the shape maintenance; it also reveals that actomyosin contractility has a role in the apical adhesion between the columnar and squamous cells.

## Discussion

The regulation and maintenance of an organ’s shape is a major outstanding question in developmental biology. The *Drosophila* wing imaginal disc serves as a powerful system for elucidating design principles of the shape formation in epithelial morphogenesis [6,7]. Yet, even simple epithelial systems such as the aforementioned wing disc are extremely complex. A tissue’s shape emerges from the integration of many biochemical and biophysical interactions between proteins, subcellular components, and cell-cell and cell-ECM interactions. How cellular mechanical properties affect tissue size and patterning of cell identities on the apical surface of the wing disc pouch has been intensively investigated [48,49]. However, less effort has focused on studying the mechanisms governing the shape of the wing disc in the cross-section. Both the significance and difficulty of such studies are due in part to the need to consider the composite nature of the material consisting of multiple cell layers and cell-ECM interactions as well as the elongated shape of columnar cells (Fig 1B).

In this study, we iterated between experiments and newly developed computational model simulations to reverse-engineer the curved profile of the larval wing imaginal disc. This effort is aligned with an overall goal to elucidate the general principles of morphogenesis. Namely, we developed a combined experimental imaging and biologically calibrated computational modeling approach to decouple the roles of actomyosin contractility, extracellular matrix tensile stress, and cell-cell interactions involved in regulating organ morphology and nuclear positioning within cells. This resulted in the detailed quantification of the distribution of nuclei along the apical-basal axis, the height of the pouch, and the curvature of the pouch along the anterior-posterior axis.

Overall, our study defined the balance of forces determining the shape of the wing imaginal disc. The central insight obtained in this work suggests that actomyosin contractility can effectively generate the curved tissue profile along the anterior-posterior axis while tension within the ECM is sufficient and necessary for preserving the bent shape even in the absence of continued actomyosin contractility once the shape is generated. Extremely high levels of tensile stresses within the ECM along the basal side of columnar would be required to generate the “dome-shaped” profile of the late 3^rd^ instar wing disc. Therefore, passive tension built up in the ECM (perhaps to be generated over time due to differential growth patterns) is unlikely to contribute significantly to shape generation, at least for the bent profile of the disc cross-section along the AP axis. Hence, our study suggests another possible mechanism that is different than differential growth hypothesis [39] to drive fold formation along the orthogonal dorsal-ventral axis. Tissue shape formation by independent mechanical systems at the apical and basal side of the cells is also reported by a subsequent report posted during the review of this manuscript [50]. Perturbation studies that chemically dissolve collagen revealed that the ECM is essential to maintain the shape of the wing disc. Therefore, it provides “shape memory” for the tissue. Comparing experimental and computational results obtained under multiple perturbations demonstrated that actomyosin contractility was effective in the generation of the bend profile of wing discs, while ECM can effectively preserve the shape of the tissue.

Our results complement a recent report on the importance of lateral and basal contractility in the wing disc in the formation of several folds along the dorsal-ventral axis [15]. In contrast, our study focused on the bent morphology along the orthogonal anterior-posterior axis. Further, our work also demonstrates a key mechanical role of asymmetrical nuclear positioning in defining the bent profile of the wing disc. Previous work has indicated that actomyosin contractility was important for the motion of mitotic cells within the wing disc [32]. Our results implicate bent profile of wing disc as well as basal actomyosin contractility in biasing all nuclei of the wing disc toward the apical surface. As the nucleus is stiffer than the cytoplasm, the positioning of nuclei near the apical surface may contribute to the formation of the shape of the disc.

Previous studies found that the peripodial membrane, a squamous epithelium that sits on top of the wing disc pouch, is connected to the pouch through microtubule-rich cellular extensions [19]. However, the implications of this interlayer connection are still not fully understood. Here, we found that adhesion between the two layers keeps the pouch and peripodial membrane together even under extreme morphological perturbations such as chemical digestion of the extracellular matrix. This provides general insight into latent interactions that ensure tissue integrity. Actomyosin contractility is needed to be maintained in this case for the two layers to remain adherent in localized regions such as the dorsal-ventral compartment boundary where the distance between the two layers is minimal. Separation of these two layers occurred with dual perturbations of collagenase and ROCK inhibition. This is likely due to the need of basally directed actomyosin contractility to keep pouch cells in close apposition to the peripodial membrane. Thus, adhesion between the apical surfaces of intra-organ layers depends on the maintenance of ROCK-driven actomyosin activity in the tissue to prevent delamination. Consequently, the spatiotemporal patterning of actomyosin contractility provides additional, indirect roles of actomyosin activity in maintaining tissue integrity that become apparent only through a series of perturbation experiments. From the collagenase-treatment experiment and the corresponding simulation, we also uncovered the pulling force from squamous cells over the columnar cells through marginal cells as a third mechanism that acts in an antagonistic way to the ECM tensile stress and actomyosin contractility. This pulling force results in the wing disc inversion along the AP cross-section of the tissue observed when the ECM is removed by collagenase.

Genetic inhibition of Rho1, a key regulator of actomyosin contractility, confirmed the role of this pathway in defining tissue curvature. The analysis further revealed a qualitative decrease in F-actin levels in basolateral regions of the columnar cells. However, as previously noted [13], the height of the cells is increased when Rho1 activity is increased. We found high levels of activated myosin near the basal and apical surfaces of columnar cells showing this height increase compared to controls (Fig 6). This suggests that other regulators of Myosin II are involved in specifying the asymmetry in actomyosin contractility. Local contractility at the apical and basal surface may play important roles in cell height control, which computational simulations could help to address in the future. Other Rho family GTPases such as Cdc42 have been implicated in cell height regulation [51], and their roles in defining subcellular contractility and overall tissue shape require further elucidation.

Larval wing disc development serves to generate the precursor to the fully formed wing. Wing morphogenesis occurs during subsequent pupal development and requires significant remodeling of the extracellular matrix [52,53]. Therefore, the mechanisms tested in this paper explain the emergence of the key initial input geometry of the wing disc leading up to eversion and wing morphogenesis occurring during pupal development. Lastly, a combination of the multi-scale subcellular element modeling environment described in this paper and specially designed experiments can be readily extended to generate and test hypothesized novel mechanisms of the wing disc eversion process and other epithelial systems, including organoids, that consist of several cellular and ECM layers.

## Materials and methods

### Fly culture and developmental staging

Details on fly husbandry and developmental staging, wing disc immunohistochemistry and imaging are provided in S1 Text, S2 Text and S3 Text.

### Tissue surface and nuclei segmentation

In-house Matlab scripts were written to facilitate tissue orientation and segmentation. This program allows the user to trace the long axis and provides the corresponding cross section on which the user segments the apical and basal surfaces. Nuclei within these cross-sections were automatically segmented (Fig A in S4 Text). Once surface and nuclei segmentations were performed, the program calculates the local curvature of the basal surface as described in Fig 2F as well as the fractional nuclei positions (Fig 2B).

### Computational model

**Fig 10:**
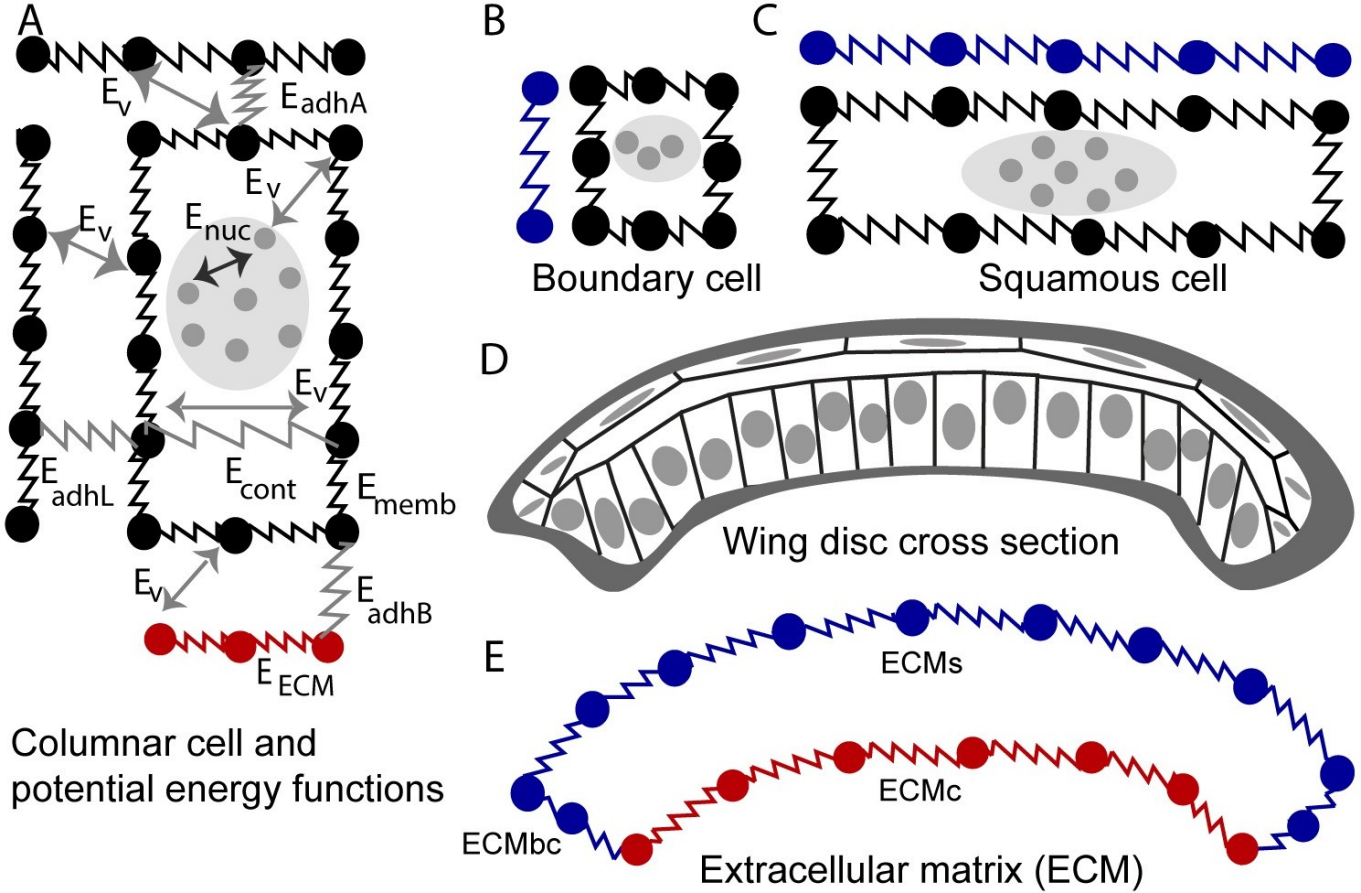
Diagram of the subcellular element (SCE) model of the cross-sectional profile of the wing along the major anterior-posterior axis. **(**A) Columnar cell submodel with its potential energy functions describing interactions between adjacent cells, intracellular interactions, and cell-ECM interactions. (B) Marginal boundary cell submodel. (C) Squamous cell submodel. (D) Diagram of the cross-sectional profile of the wing along the anterior-posterior axis which includes columnar cells, boundary cells, squamous cells, and ECM. (E) Diagram of the submodel of the ECM divided into separate sections: ECMc, ECM associated with columnar cells in the wing pouch; ECMbc, ECM associated with the marginal boundary cells at the lateral region of the disc, and ECMs, ECM associated with squamous cells.

A novel multi-scale subcellular element (SCE) model was developed and calibrated using experimental data to simulate the cross-sectional profile of the wing along the anterior-posterior axis. The model decouples direct and indirect effects of actomyosin-generated forces, nuclear positioning, passive tension in the extracellular matrix (ECM), and adhesion in shaping *Drosophila* wing imaginal discs. Model describes columnar cells (Fig 10A), boundary cells (Fig 10B) and squamous cells (Fig 10C). The model includes three types of nodes (nucleus nodes, membrane nodes and ECM nodes) to represent mechanical properties of the cell nucleus, cell membrane, and ECM, with interactions between nodes prescribed by different energy functions. Positions of all nodes describing different sections of the ECM and different types of cells are updated by using different potential energy functions listed in Table 1 and based on Langevin equations:

$$C_{nuc}{\dot{x}}_{nuc}=-(\nabla E_{nuc}+\nabla E_{v})$$ (1)

$$C_{memb}{\dot{x}}_{memb}=-(\nabla E_{memb}+\nabla E_{cont}+\nabla E_{v}+\nabla E_{vol}+\nabla E_{adhL}+\nabla E_{adhB}+\nabla E_{adhA})$$ (2)

$$C_{ECM}{\dot{x}}_{ecm}=-(\nabla E_{ecm}+\nabla E_{v}+\nabla E_{adhB}).$$ (3)

Langevin equations assume that cell motion occurs in the overdamped regime which is a valid assumption for cellular modeling in most biological systems [54–57]. Each Langevin equation includes a constant damping term. Other forms of damping, such as dissipative frictional force damping [58] which represents shear viscosity force between membranes of cells and between membranes of cells and ECM are neglected in the current study.

**Table 1:** Potential energy functions used in the subcellular element model.

Energy term	Type	Physical representation
*E*_*v*_	Morse	Volume exclusion
*E*_*nuc*_	Morse	Size of nucleus
*E*_*adhL*_	Spring	E-cadherin (cell-cell adhesion)
*E*_*adhB*_	Spring	Integrin (cell-ECM adhesion)
*E*_*adhA*_	Spring	Adhesion between columnar and squamous cells
*E*_*cont*_	Spring	Actomyosin contractility inside the cells and beneath the nucleus
*E*_*memb*_	Spring	Actomyosin contractility at the cell cortex and membrane stiffness
*E*_*ecm*_	Spring	Extracellular matrix stiffness
*E*_*vol*_	Lagrange multiplier	Volume conservation of cytoplasm for each cell

The right-hand side (RHS) of the Eq 1, describing of motion for nucleus nodes, includes two Morse potential energy functions. *E*_*nuc*_ (shown in Fig 10 and Table 1) represents interaction forces between nucleus nodes. Values of coefficients in *E*_*nuc*_ (Table A in S7 Text) are set such that the desired volume of the nucleus is achieved, and, meanwhile, the nucleus nodes stay sufficiently close to each other within the same cell (Fig 10A). *E*_*v*_, representing interactions between nucleus nodes and membrane nodes, prevents nucleus nodes from passing or overlapping with the membrane nodes. At the same time, such interactions generate forces causing nucleus motion. The motion of each nucleus described by motion of a cluster of its corresponding nodes is an emergent property of the model simulation. For example, when basal membrane nodes of columnar cells contract, *E*_*v*_ induces the motion of nucleus nodes toward the apical side of columnar cells (as was observed in experiments [32]) and *E*_*nuc*_ makes sure that all nucleus nodes move together. (Also, see the inset—zoom-in view to the Fig 5A’ of interacting columnar cells nodes and nucleus nodes.). In general, *E*_*v*_ in (Eqs 1–3) describes volume exclusion between different types of nodes.

Eq 2 describes the motion of membrane nodes representing cortical stiffness of the cells and lipid bilayer membranes of the cells. The RHS of the Eq 2 consists of seven potential energy functions. *E*_*memb*_ is the spring potential energy function between adjacent membrane nodes of the same cell (shown in Fig 10). Spring coefficient of *E*_*memb*_ represents the level of stiffness of the lipid bilayer of the membrane and actomyosin at the cortex of the cell. *E*_*adhL*_ is the pairwise spring potential describing the interaction between membrane nodes of neighboring cells along the lateral sides. *E*_*adhB*_ is the spring potential energy function between basal membrane nodes and ECM nodes representing the force provided by integrin, a transmembrane protein, as cells adhere to the ECM. *E*_*adhA*_ is the potential energy function of springs between apical membrane nodes of squamous and columnar cells representing adhesion between these two types of cells [19]. *E*_*cont*_ is the spring potential energy function connecting the membrane nodes located at the opposite lateral sides beneath the nucleus of one columnar cell. The coefficient of the *E*_*cont*_ represents the level of basal actomyosin contractility (*k*_*cont*_). Finally, *E*_*vol*_ is the Lagrangian multiplier potential energy function applied to all membrane nodes to make sure the volume of each cell is conserved (see S5 Text for more details).

Eq 3 describes the motion of ECM nodes representing ECM surrounding the basal side of epithelial cells. The RHS of the Eq 3 includes three potential energy functions. *E*_*ecm*_ is the spring potential energy function between adjacent nodes of the ECM. The coefficient of the *E*_*ecm*_ represents stiffness of the ECM mainly provided by the collagen. *E*_*adhB*_ and *E*_*v*_ are the same terms as included in Eqs 1 and 2. ECM nodes are subdivided into three types: nodes connected with columnar cells, nodes connected with boundary cells and boundary cells, which are denoted by ECMc, ECMbc and ECMs, respectively. To solve Eqs 1–3, the explicit Euler method was applied, and model simulations were carried out on a cluster of graphical processing units (GPUs) to accelerate the computation. (For more detailed information about discretization and GPU implementation, please see [57].)

Cell-cell adhesion and cell-ECM adhesion model parameters were calibrated using experimentally obtained values of adhesion for epithelial cells [57] since it was reported that the level of cell-cell adhesion for epithelial cells is in the same range as the cell-ECM adhesion [59]. The size and number of cells in the model were calibrated using experimentally observed shapes of epithelial cells (Table 2). The ranges of all model parameters are provided in S7 Text.

**Table 2:** Calibration of the model using experimental data over 96 hours of development of the cross-sectional profile of the wing along the anterior-posterior axis.

Properties	Experiment (this work)	Simulation
Number of columnar cells	60–70	65
Columnar cells width	2–3 μ*m*	2.5 μ*m*
Columnar cells height	25–35 μ*m*	25 μ*m*
Number of squamous cells	10–15	10
Squamous cells width	15–18	16
Squamous cells height	3–5	4
Nucleus distribution of columnar cells	60% ± 16%	70% ± 16%

**Fig 11:**
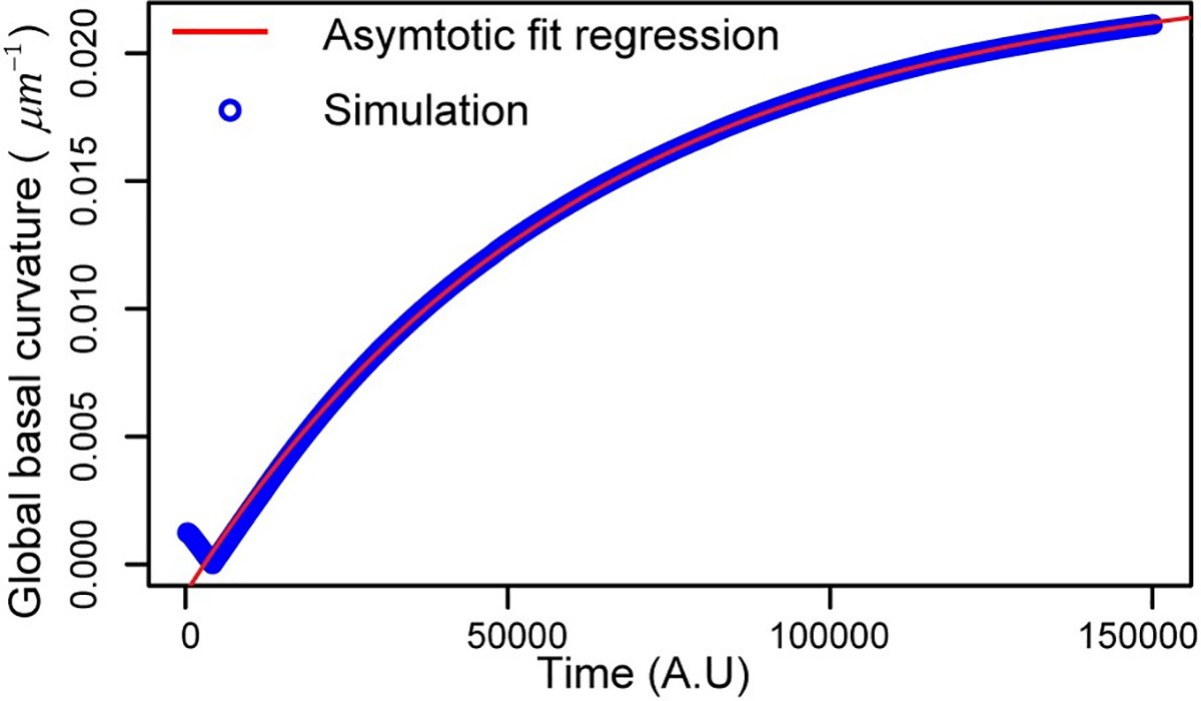
Temporal evolution of global curvature (blue) and its asymptotic fitting regression curve (red). Simulation leading to a nearly steady state shape in the wild-type simulation. Simulation runs on a dedicated GPU for 192 hours.

We checked for convergence in model simulations by plotting global curvature of the wing disc tissue versus time and quantifying the change in curvature between two successive time steps. A flat sheet of cells was used as the initial condition with zero global curvature (Fig 4A and Fig 5A). The parameters for the initial shape of the tissue are given based on measurements of different properties of the cross-section of wing disc by our group and it is shown in Table 2. Simulation is run for 150,000 times steps (arbitrary units, A.U.) with time step size of 0.002 AU, which is plotted in Fig 11. The asymptotic fitting regression was also plotted to quantify the final value of the global curvature. The asymptotic fit regression model fits well with the simulation results, showing that reliable convergence is obtained (Fig 11). The code for this computational framework was developed in CUDA C++, runs on GPU clusters, and it is available on GitHub: https://github.com/AliNemat/EpiScale_CrossSection.git.

## Supporting information

S1 Text Additional fly culture details. (PDF)

S2 Text Wing disc immunohistochemistry and mounting. (PDF)

S3 Text Mounting of stained wing discs. (PDF)

S4 Text Additional image processing methods. (PDF)

S5 Text Additional description of the computational approach. (PDF)

S6 Text Latin hypercube sampling (LHS) method and sensitivity analysis. (PDF)

S7 Text Computational model parameters. (PDF)

S1 Video Computational model for bent profile formation of cross section of imaginal wing disc along major axis, and subsequent perturbation by removal of extra cellular matrix and basal actomyosin contractility, while maintaining apical adhesion between columnar and squamous cells. (MP4)

S2 Video Experimental data for perturbation of imaginal wing disc along major axis by removal of extra cellular matrix and actomyosin contractility. (AVI)
