# Supplementary material for: Epithelial organ shape is generated by patterned actomyosin contractility and maintained by the extracellular matrix
Source: PLoS Comput Biol. 2020 Aug 20;16(8):e1008105. doi: 10.1371/journal.pcbi.1008105 (PMC7480841; doi:10.1371/journal.pcbi.1008105)
Supplement: S1 Text — (PDF) [file pcbi.1008105.s001.pdf]

S1 Text: Additional fly culture details

Flies were raised at 25 °C and a twelve-hour light cycle unless specified otherwise. For all staging experiments, adult flies were used three days after pupal eclosion. These adults were placed in cages containing grape agar plates and yeast paste for four hours. Eggs from this collection period were allowed to mature on the agar plates for 24 hours after which first instar larva were transferred to new agar plates. The time used for the developmental age was determined from the midpoint of the egg collection period. Fly stocks include: The *Drosophila melanogaster* line Oregon-R was used in all staging experiments (Fig A). Vkg:GFP expressing flies were used for imaging of the ECM. MS1096-Gal4 were used to generate results in Figure 6.

Live images were collected from discs cultured in Grace's medium (ThermoFisher, 11595030) with low ecdysone (Sigma, H5142) [1]. Live imaging experiments were performed for up to four hours with 100-200, 1 μm slices taken across the z-direction every 5 minutes. Note that the imaging conditions required for live-imaging do not provide as fine resolution as for fixed images. 1 mM Y-27632 (Selleck Chemicals LLC, S1049) was used for ROCK inhibitor experiments [2,3]. 3 mg/ml Collagenase (Worthington Biochemical, LS004194) was used for ECM inhibition experiments. 4 μM Latrunculin A (Sigma-Aldrich, L5163) was used to inhibit actin during live imaging [4]. Finally, 1 μl/ml of Cell Mask (ThermoFisher, C10046) was used to visualize the shape of the disc during treatment.

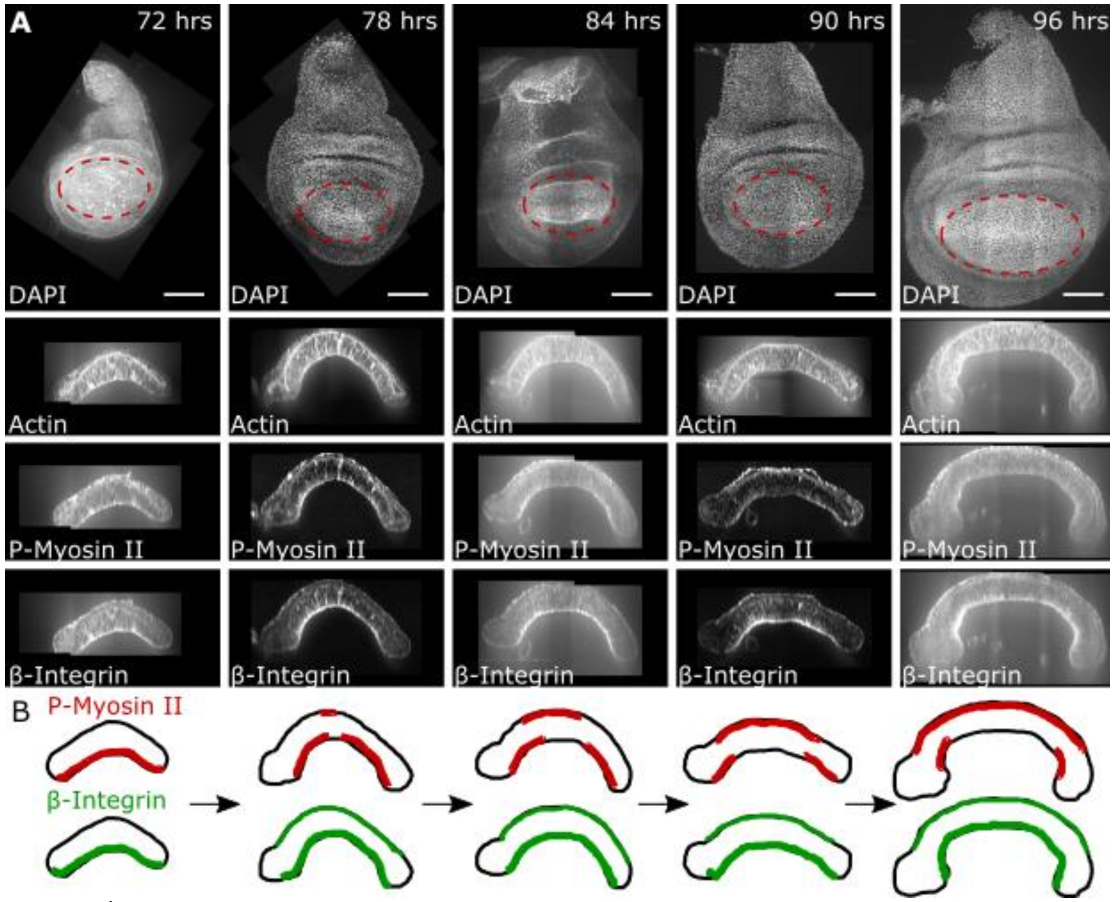

Fig A. 3rd instar wing discs exhibit pronounced curvature along the anterior-posterior axis. A) Wing disc development from 72 to 96 hours AEL. The first row shows z-projections of nuclear signals (DAPI). Rows 2-4 are cross-sections along the anterior-posterior axis showing F-actin (phalloidin), P-Myosin II, and $\beta-\mathrm{integrin}$. Scale bars are 50 μm. **B)** Schematic showing asymmetric of P-Myosin II and β-Integrin for each timepoint in A. The genotype for Fig A is Ore R.

References:

1. Dye NA, Popović M, Spannl S, Etournay R, Kainmüller D, Ghosh S, et al. Cell dynamics underlying oriented growth of the Drosophila wing imaginal disc. Development. 2017 Jan 1;dev.155069.
2. Landsberg KP, Farhadifar R, Ranft J, Umetsu D, Widmann TJ, Bittig T, et al. Increased Cell Bond Tension Governs Cell Sorting at the Drosophila Anteroposterior Compartment Boundary. Curr Biol. 2009 Dec 1;19(22):1950–5.
3. Farhadifar R, Röper J-C, Aigouy B, Eaton S, Jülicher F. The Influence of Cell Mechanics, Cell-Cell Interactions, and Proliferation on Epithelial Packing. Curr Biol. 2007 Dec 18;17(24):2095–104.
4. Sui L, Alt S, Weigert M, Dye N, Eaton S, Jug F, et al. Differential lateral and basal tension drive folding of Drosophila wing discs through two distinct mechanisms. Nat Commun [Internet]. 2018 Nov 5 [cited 2019 Jun 17];9. Available from: https://www.ncbi.nlm.nih.gov/pmc/articles/PMC6218478/
