# Supplementary material for: Epithelial organ shape is generated by patterned actomyosin contractility and maintained by the extracellular matrix
Source: PLoS Comput Biol. 2020 Aug 20;16(8):e1008105. doi: 10.1371/journal.pcbi.1008105 (PMC7480841; doi:10.1371/journal.pcbi.1008105)
Supplement: S2 Text — (PDF) [file pcbi.1008105.s002.pdf]

S2 Text: Wing disc immunohistochemistry and mounting.
Wing imaginal discs from 3rd instar *Drosophila* larva were dissected in intervals of 20 min. Dissected wing discs were then fixed in ice cold 10% neutral-buffered formalin (NBF) for 20 min in PCR tubes. Immediately following fixation, wing discs were rinsed three times with fresh PBT (PBS with 0.03% v/v Triton X-100). Tubes containing wing discs were then placed on a nutator for 10 minutes at room temperature and then rinsed again with PBT; this step was repeated for a total of three nutation/rinsing intervals. PBT from the final rinse was removed and 200 µL of 5% normal goat serum (NGS) in PBS was added to each PCR tube. Tubes were then placed on a nutator for 30 minutes at room temperature. NGS was then removed and 200 µL of a primary antibody mixture, prepared in 5% NGS solution, was added. Tubes were then placed on a nutator at 4˚C overnight. Three quick rinses were then performed as was done after fixation. Tubes were placed on a nutator for 15 minutes followed by PBT rinsing; repeated for a total of three nutation/rinsing intervals. PBT from the final rinse was then removed and 200 µL of a secondary antibody mixture, prepared in 5% NGS solution, was added. Tubes were then placed on a nutator for 2 hours at room temperature. Three quick rinses were then performed as before. Tubes were then placed on a nutator for 20 minutes at room temperature and then rinsed with PBT for a total of three intervals. Wing discs were then either kept in tubes at 4˚C or mounted immediately.
