# Supplementary material for: Epithelial organ shape is generated by patterned actomyosin contractility and maintained by the extracellular matrix
Source: PLoS Comput Biol. 2020 Aug 20;16(8):e1008105. doi: 10.1371/journal.pcbi.1008105 (PMC7480841; doi:10.1371/journal.pcbi.1008105)
Supplement: S3 Text — (PDF) [file pcbi.1008105.s003.pdf]

S3 Text: Mounting of stained wing discs

Wing discs were mounted between 24 x 60 mm and 22 x 22 mm glass cover slips with VECTASHIELD® (Vector Laboratories). For images in which the natural curvature of the wing disc was desired, Scotch tape was used as a spacer between the two cover slips. Clear nail polish was used to seal in VECTASHIELD® and to adhere cover slips together.
