# Supplementary material for: Epithelial organ shape is generated by patterned actomyosin contractility and maintained by the extracellular matrix
Source: PLoS Comput Biol. 2020 Aug 20;16(8):e1008105. doi: 10.1371/journal.pcbi.1008105 (PMC7480841; doi:10.1371/journal.pcbi.1008105)
Supplement: S4 Text — (PDF) [file pcbi.1008105.s004.pdf]

S4 Text: Additional image processing methods.

Proper orientation is critical for determining tissue thickness, since misalignment in one plane affects measurements taken from images of orthogonal planes. To prevent measurement errors associated with misalignment, we developed a pipeline for quick image orientation and surface segmentation (Fig A). This pipeline was used to extract apical and basal surfaces along the major and minor pouch axes. We chose global curvature as a simple metric to quantify curvature development during wing disc growth. Global curvature is calculated by fitting an arch of a circle through a set of data points and taking the inverse of the circle's radius. Global curvatures were calculated for the segmented apical and basal surfaces (Fig A).

To quantify the nuclear position in the cross-section of wing discs, we developed a pipeline to segment nuclei from images of DAPI (DNA marker) stained tissues (Fig B). This pipeline implements various image processing steps followed by calculation of the centroid of each nucleus. The statistics obtained by this quantification are shown in Fig B.

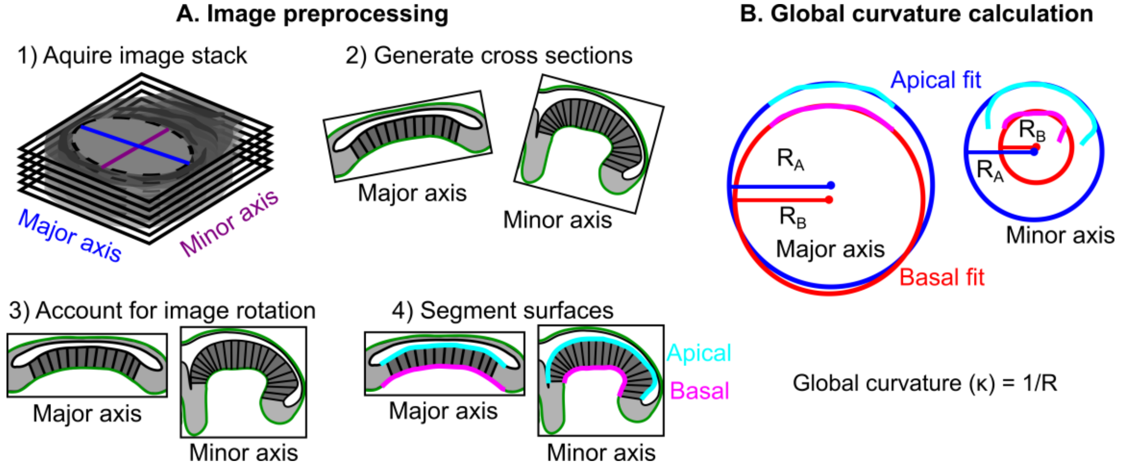

Fig A. Description of the pipeline developed to extract the cross-section of wing discs and quantification of global curvature as the metric to compare different phenotypes. A) Pipeline to extract apical and basal surfaces along the major and minor pouch axes. **B)** Global curvature is obtained by fitting an arch of a circle through a set of data points selected from the surfaces.

To quantify the difference in tissue's thickness due to ECM degradation, distances were extracted from the apical to basal surface through each segmented nuclei. Collagenase treatment experiments were performed to decouple the mechanical contribution of the extracellular matrix from all other tissue components. Collagenases are enzymes that break the peptide bonds in collagen, a structural protein of the ECM. Viking (Vkg) is a subunit of collagen IV, and we expressed Vkg::GFP in the wing disc to visualize collagen within the epithelium. We incubated wing discs in live culture media containing 3.4 mg/ml collagenase for 1 hour. Before media preparation, collagenase was dissolved in Phosphate-buffered saline (PBS). To ensure that differences between control and treated wing discs were due to collagenase, we added an equivalent volume of PBS to the control media. We found that thickness differences were significant between the untreated and collagenase treated conditions (Fig C).

Fig D shows the distribution of phosphorylated Myosin II in cross-section, as well as in individual planes encompassing the squamous peripodial cells, the columnar pouch cells near the apical surface, and near the basal surface of the columnar pouch cells.

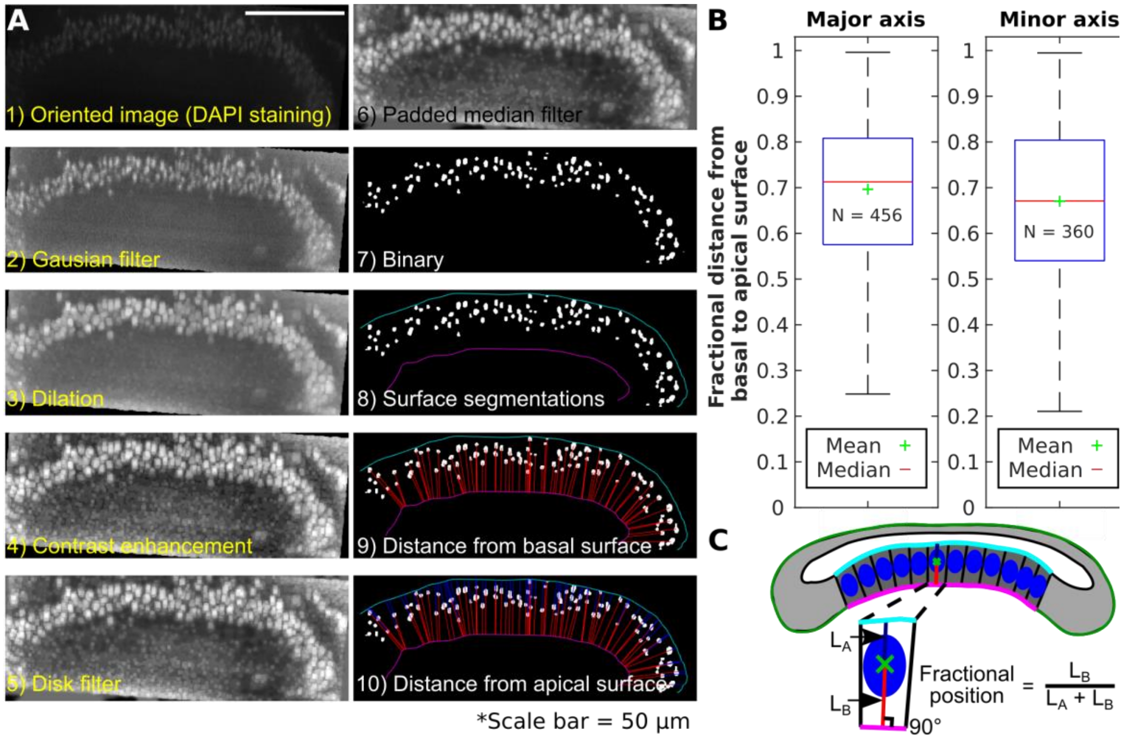

Fig B. Quantification of apical nuclear bias. A) The pipeline implements various image processing steps (1-6) followed by extracting the centroid of each nucleus (7). Lines perpendicular to the basal surface, obtained in (8), are then generated starting from each nuclear centroid (9). The distances from each centroid to the apical and basal surfaces are then calculated as the fractional positions of nuclei (10). **B)** Boxplots of the fractional distances of nuclei from basal to apical surfaces. **C)** Definition of fractional position.

Finally to calculate the Menger curvature for the intensity correlations of experimental data for the major axis of wing disc shown in Fig 2G and Fig 2H, a region of interest was manually selected using an open source MATLAB package 'roispline' [1]. A curve defining the apical surface of wing disc was then separated from the wing disc boundary points defined by the fitted spline. An equally spaced fixed number of boundary points were then selected from the curve as indicated by a red dot in Fig 2G. For any set of ith points, we defined Menger curvature as the reciprocal of the radius of the circle passing through the three points [2]. Fluorescence intensity around any i^{th} point was defined as the averaged integrin intensity along the curve defined between the $i\text{-}1^{th}$ and $i\text{+}1^{th}$ point. The circle is fit using an inbuilt MATLAB function 'circumcenter'. Then to calculate local curvature points of the wing disc boundary were selected interactively using custom built MATLAB functions. Between any two consecutive points, a natural cubic spline was fit following which the segment defining apical boundary was separated from the wing disc. The whole methodology is similar to one described above. We then used the spline S_i defined between points X_i and X_{i+1} to interpolate sufficient number of points for estimation of derivatives and double derivatives which are further used for curvature calculation.

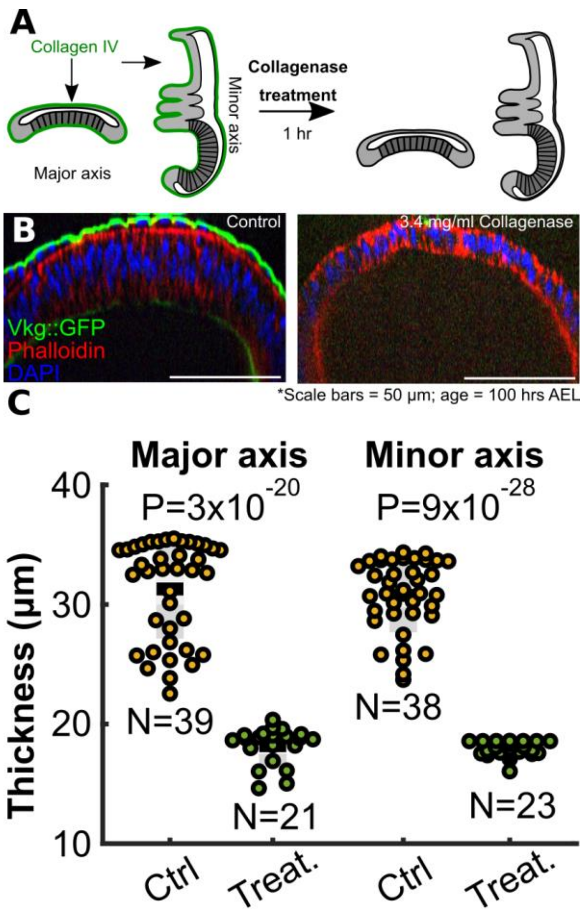

Fig C. Chemically based ECM removal. ECM degradation by collagenase treatment (A) resulted in a visually distinct decrease in thickness of columnar epithelium (B) as quantified in (C).

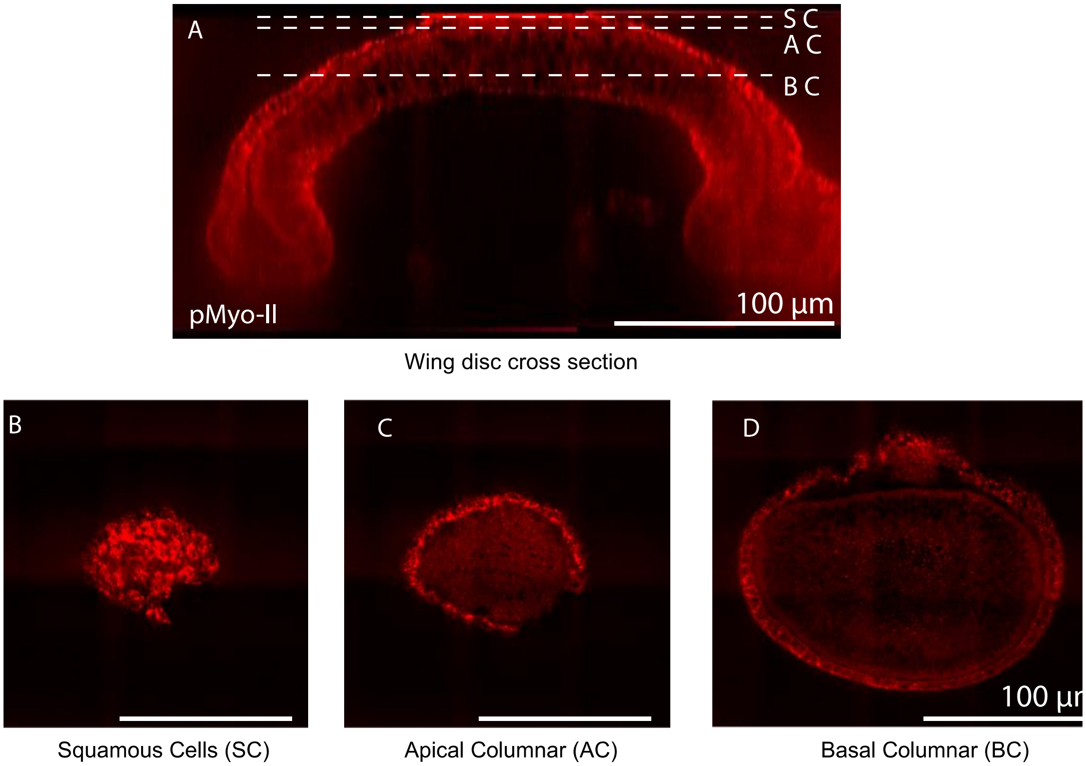

Fig D. Distribution of phosphorylated Myosin-II (pMyo-II) in cross-section and in individual z-slices of wing discs. **A)** pMyo-II distribution in the cross-section of the wing disc along the anterior-posterior axis, **B)** a single z-slice covering the squamous peripodial cells. **C)** pMyo-II distribution near the apical surface of the pouch cells, **D)** the basal surface of pouch cells.

.

References:

1. Select ROI in image using spline - File Exchange - MATLAB Central [Internet]. [cited 2020 Jan 15]. Available from: https://www.mathworks.com/matlabcentral/fileexchange/12530-select-roi-in-image-using-spline
2. Léger J-C. Menger curvature and rectifiability. Ann Math. 1999;149:831–869.
