# Supplementary material for: Epithelial organ shape is generated by patterned actomyosin contractility and maintained by the extracellular matrix
Source: PLoS Comput Biol. 2020 Aug 20;16(8):e1008105. doi: 10.1371/journal.pcbi.1008105 (PMC7480841; doi:10.1371/journal.pcbi.1008105)
Supplement: S5 Text — (PDF) [file pcbi.1008105.s005.pdf]

S5 Text: Additional description of the computational approach.
The Lagrange multiplier energy function E_{vol} is used to guarantee the conservation of volumes of individual cells (mainly cytoplasm) as follows:

$$E_{vol} = k_{vol}(\Omega - \Omega_0)^2, \quad \text{(S5-1)}$$

where Ω is the current volume of the cell and Ω_0 is the prescribed cellular volume. The coefficient k_{vol} determines the strength of the enforcement of the volume constrain. Following previous studies [1,2], we choose a specific value k_{vol} (Table A in S1 Text) such that the cell volume remains conserved in all simulations with 90% accuracy.

Eqs. 1-3 in the main text describe motion of different types of nodes, which is determined by the gradient of the corresponding potential energy function. For example, E_v , a Morse energy potential function determining volume exclusion, has the following form:

$$E_v = U_v exp\left(\frac{-|\boldsymbol{x}_i - \boldsymbol{x}_j|}{\zeta_v}\right) - W_v exp\left(\frac{-|\boldsymbol{x}_i - \boldsymbol{x}_j|}{\gamma_v}\right) \quad \text{(S5-2)}$$

Then the gradient of the E_v is as follows:

$$\nabla E_v = \left(\frac{-U_v}{\zeta_v} exp\left(\frac{-|\boldsymbol{x}_i - \boldsymbol{x}_j|}{\zeta_v}\right) + \frac{W_v}{\gamma_v} exp\left(\frac{-|\boldsymbol{x}_i - \boldsymbol{x}_j|}{\gamma_v}\right)\right)\frac{\boldsymbol{x}_i - \boldsymbol{x}_j}{|\boldsymbol{x}_i - \boldsymbol{x}_j|}, \quad \text{(S5-3)}$$

where U_v , ζ_v , W_v, and γ_v are Morse coefficients, and $\boldsymbol{x}_i$ and $\boldsymbol{x}_j$ are vectors describing locations of nodes *i* and *j*. All vectors describing locations of nodes are start from zero. Minus of gradient of potential energy is equal to force and Eq. S5-3 describes the impact of the force applied to node i by node j due to E_v.

By using the SCE model developed above, we investigated roles of ECM and basal actomyosin contractility in bending the wing disc tissue along the AP axis. Regarding the role of ECM, we compared the modeling results obtained with differential tensile stresses in the ECM which is modeled by assuming different values for $L_{0_{ECMc}}$ and $L_{0_{ECMs}}$ (see Fig 10). Tensile forces applied to the ECMc and ECMs are defined as follows:

$$F_{ECMc} = k_{ECM}(L - L_{0_{ECMc}}), \quad \text{(S5-4)}$$
$$F_{ECMs} = k_{ECM}(L - L_{0_{ECMs}}). \quad \text{(S5-5)}$$

Since k_{ECM} is identical for ECMc and ECMs, lower value of $L_{0_{ECMc}}$ compared with $L_{0_{ECMs}}$ leads to higher initial tensile stress in ECMc compared with ECMs.

The role of actomyosin contractility was investigated by varying the stiffness of the springs connecting lateral nodes below nuclei of columnar cells in the model (Fig. 10). As the spring

stiffness is increased, the tendency of columnar cells to squeeze beneath the nuclei is also enhanced based on the following equation

$$F_{cont} = k_{cont}(L_{cont} - L_{0_{cont}}). \quad \text{(S5-6)}$$

To identify each bending tissue mechanism, we separately varied the ratio between F_{ECMc} and F_{ECMs} and F_{cont}. The resulting tissue shape evaluated in terms of overall curvature, relative nuclear positions in individual cells and the height of columnar cells was compared with the experimental data as shown in Fig 4 and Fig 5.

References:

1. Pivkin IV, Karniadakis GE. Accurate coarse-grained modeling of red blood cells. Phys Rev Lett. 2008;101(11):118105.
2. Li J, Dao M, Lim CT, Suresh S. Spectrin-level modeling of the cytoskeleton and optical tweezers stretching of the erythrocyte. Biophys J. 2005;88(5):3707–3719.
