# Supplementary material for: Epithelial organ shape is generated by patterned actomyosin contractility and maintained by the extracellular matrix
Source: PLoS Comput Biol. 2020 Aug 20;16(8):e1008105. doi: 10.1371/journal.pcbi.1008105 (PMC7480841; doi:10.1371/journal.pcbi.1008105)
Supplement: S6 Text — (PDF) [file pcbi.1008105.s006.pdf]

S6 Text: Latin hypercube sampling (LHS) method and sensitivity analysis.

The Latin hypercube sampling (LHS) method was applied to perform the sensitivity analysis of the bending shape of tissues with respect to multiple parameters in the computational model [1]. LHS method is one of the most efficient sampling methods for sensitivity analysis, especially when the number of parameters is large [2]. In particular, we chose average diameter of the cellular nuclei, the ratio between the tension of ECM connecting with columnar cells and squamous cells (F_{ECMc} $/F_{ECMs}$), and the level of actomyosin contractility below nuclei in columnar cells (k_{cont}) as the inputs for the sensitivity analysis. In the output, we compared the global curvature of the basal side of columnar cells, mean position of nuclei in columnar cells and the mean height of columnar cells. Minimum and maximum values for each of these three parameters are chosen using LHS method to obtain final temporal global curvature. In the LHS sensitivity analysis, the range of each parameter was divided into 10 bins and exactly 10 samples are chosen following the uniform random distribution such that there is exactly one sample selected from each bin for every parameter. Result of 10 sets of parameters obtained by this sampling method is shown in Fig A. The code is available on github: https://github.com/AliNemat/LatinHyperCube.git.

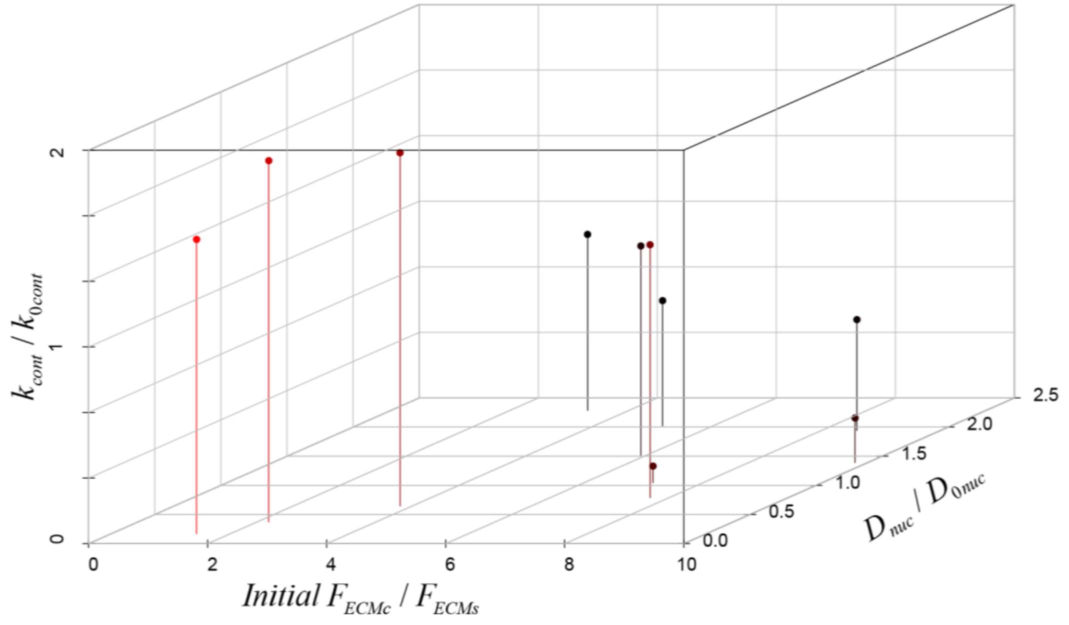

Fig A. Hypercube sampling method. Nucleus diameter, ratio of the tensions of ECM connected with the columnar cells and squamous cells, and actomyosin contractility beneath nuclei are chosen as the input parameters

After obtaining sampling parameter set using LHS method, we applied partial correlation coefficient method [1] to assess the relation between three input parameters and three output quantities measured from the computational model simulations. In particular, the global curvature was calculated by using best circle fitting method which was also used in image analysis of experimental data (Fig A in S4 Text). The average height of columnar cells is calculated by the average distance between apical and basal nodes of columnar cells. Finally, the average nuclear position in columnar cells are calculated as below

$$\overline{loc}_{nuc}(\%) = \frac{1}{n}\sum_{i=1}^{n}\frac{|\boldsymbol{x}_{nuc}(i) - \boldsymbol{x}_{bas}(i)|}{|\boldsymbol{x}_{api}(i) - \boldsymbol{x}_{bas}(i)|} \quad \text{(S6-1)}$$

where n is the number of columnar cells, $\boldsymbol{x}_{nuc}$ is the vector defining the position of the nuclear center of each cell, $\boldsymbol{x}_{bas}$ is the vector defining the basal location of each columnar cell and $\boldsymbol{x}_{api}$ is the vector defining the apical location of each columnar cell. In the partial correlation coefficient method, the relationship between an input parameter and an output measurement is characterized through removing the linear correlation between this output measurement and all other input parameters. The inputs and outputs for the sensitivity analysis are listed in Table A.

Table A. Inputs and outputs in the partial correlation coefficient study.

Input parameters	Output parameters
$x_1 = x_{ten} = F_{ECMc}/F_{ECMs}$	$y_1 = y_{curv}$
$x_2 = x_{nucD} = D_{nuc}/D_{0nuc}$	$y_2 = y_{nuc}$
$x_3 = x_{cont} = k_{cont}/k_{0cont}$	$y_3 = y_h$

Then the partial correlation coefficient between $x_i$and y_j is the correlation coefficient between the residual of the input parameter, $x_i - \hat{x}_i$, and that of the output measurement, $y_j - \hat{y}_{ji}$, where

$$\hat{x}_i = c_0 + \textstyle\sum_{\substack{p=1 \\ p\neq i}}^{3} c_p x_p, \quad \text{(SI 8)}$$

$$\hat{y}_{ji} = b_0 + \sum_{\substack{p=1 \\ p\neq i}}^{3} m_{jp} x_p. \quad \text{(SI 9)}$$

The overall result of the sensitivity analysis based on partial correlation coefficient method is shown in Fig A and Fig B. Among all three input parameters, there is strong positive linear relationship between the actomyosin contractility and all three output measurements (Fig A).

In addition, nuclear diameter is negatively correlated with the global basal curvature and cell height, while the ECM tension has weak negative correlation with the cell height. The corresponding quantification of the pairwise correlation is shown in Fig B. We can see that the correlation with actomyosin contractility is highest for all three outputs and the nuclear diameter is correlated with the global curvature and columnar cell height negatively. The ECM tension has low correlation with all three outputs. These quantifications are all consistent with results shown in Fig A. Overall, the sensitivity analysis also confirmed that basal actomyosin contractility is effective in inducing tissue bending while differential passive tension built up within ECM may contribute little to the bending shape of the *Drosophila* wing disc along the anterior-posterior axis in the pouch region.

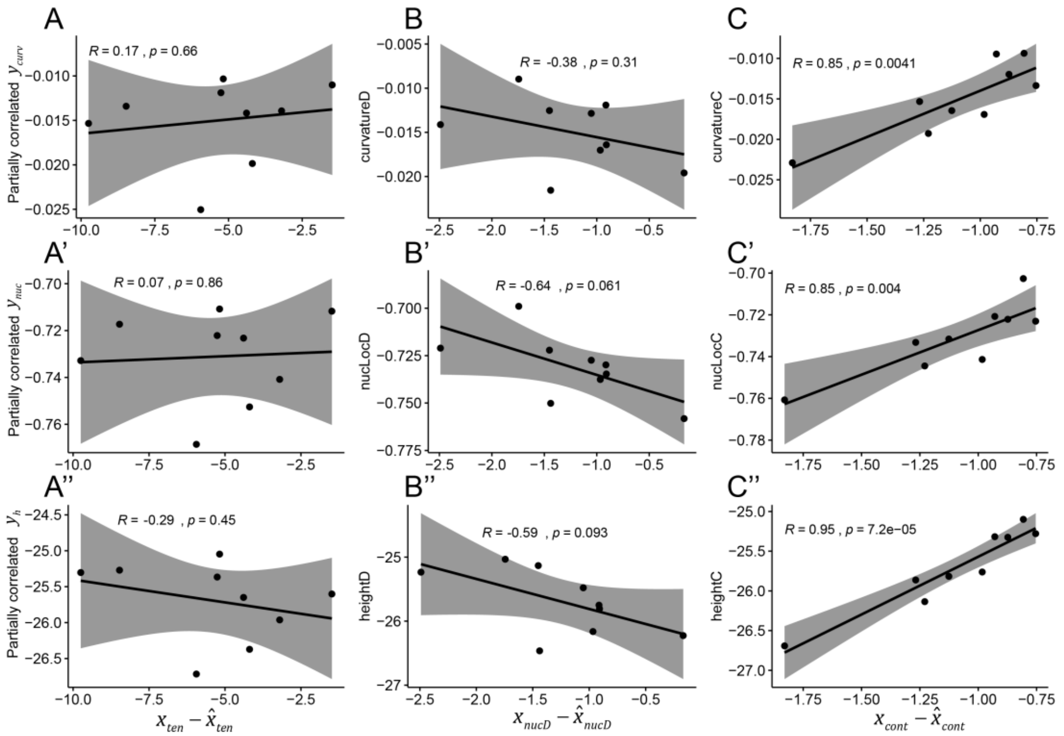

Fig A. LHS method results. Correlation between the outputs: global curvature of the tissue (A, B, C), average nucleus position (A', B', C') and average height of columnar cells (A", B", C"), and inputs: ECM relative tension (A, A', A"), nucleus average diameter (B, B', B") and basal contraction of pouch cells (C, C', C") obtained by using partial correlation coefficient method.

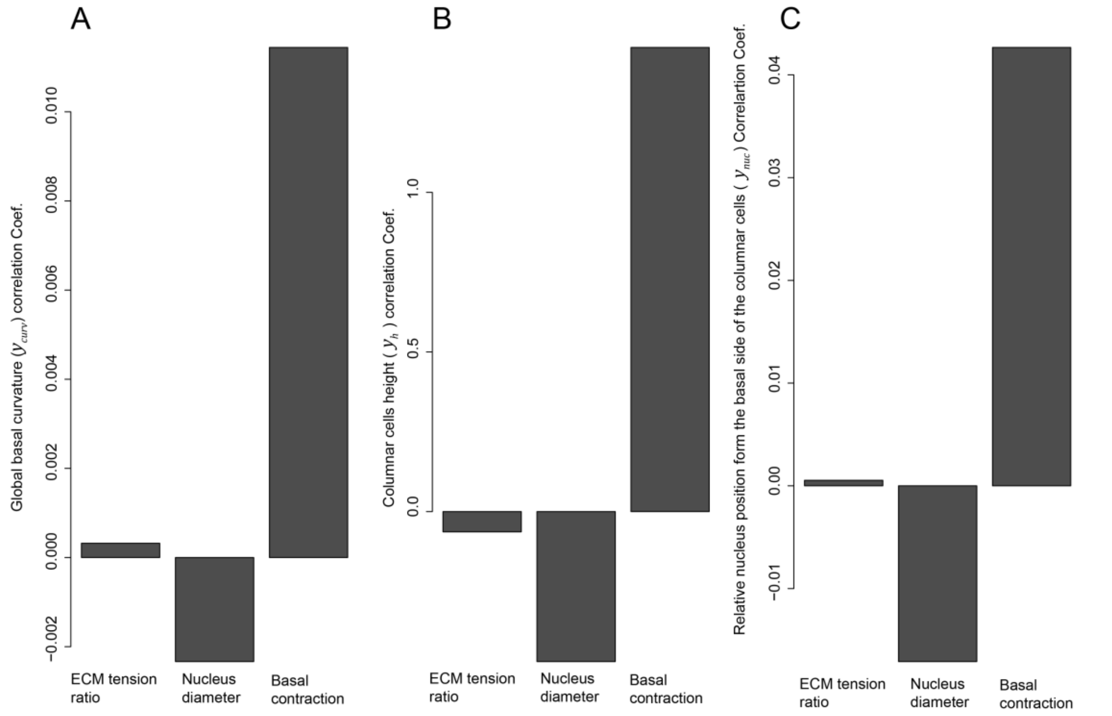

Fig B. Sensitivity of global curvature of the tissue (A), average height of columnar cells (B) and average nuclear position (C) with respect to changes in ECM relative tension, nucleus average diameter and basal contraction of columnar cells.

References:

1. Marino S, Hogue IB, Ray CJ, Kirschner DE. A methodology for performing global uncertainty and sensitivity analysis in systems biology. J Theor Biol. 2008 Sep 7;254(1):178–96.
2. Morris MD. Three Technometrics experimental design classics. Technometrics Alex. 2000 Feb;42(1):26.
