# Supplementary material for: Epithelial organ shape is generated by patterned actomyosin contractility and maintained by the extracellular matrix
Source: PLoS Comput Biol. 2020 Aug 20;16(8):e1008105. doi: 10.1371/journal.pcbi.1008105 (PMC7480841; doi:10.1371/journal.pcbi.1008105)
Supplement: S7 Text — (PDF) [file pcbi.1008105.s007.pdf]

S7 Text: Computational model parameters

Parameters used in model simulations in wild type and perturbed cases are listed in the Tables A-E.

Table A. Energy functions parameters used for dome shape profile formation in wild type simulation as shown in Fig 5A-5A' in the main text. These parameters are used in the right-hand sides of the Equations 1-3.

Energy	Definition	Interaction Type	Values	Reference
E_v	Volume exclusion	Morse	$U_v = -V_v = 7.04\, nN.\mu m$	Ref. [1]
			$\zeta_v = \mathrm{v} = 0.375\ \mu m$	
			$L_{vMax} = 0.78125\ \mu m$	
E_{nuc}	Size of nuclear	Morse	$U_{nuc} = 12.8\, nN.\mu m$	This work
			$\zeta_{nuc} = 0.224\ \mu m$	
			$V_{nuc} = 9.6\, nN.\mu m$	
			$\gamma_{nuc} = 3.36\ \mu m$	
			$L_{nucMax} = 3.5\ \mu m$	
E_{adhL}	E-cadherin	Spring	$k_{adhL} = 200\, nN/\mu m$	Ref. [1]
E_{adhB}	Integrin	Spring	$k_{adhB} = 400\, nN/\mu m$	Ref. [1,2]
E_{adhA}	Adhesion between columnar and squamous cells	Spring	$k_{adhA} = 20\, nN/\mu m$	This work
			$L0_{adhA} = L0_{adhB} = L0_{adhL} = 0.0625\ \mu m$	
E_{cont}	Basal actomyosin contractility	Spring	$k_{cont} = 9\, nN/\mu m$	Result of model calibration
			$L0_{cont} = 0.03125\ \mu m$	
E_{memb}	Membrane stiffness	Spring	$k_{memb} = 1800\, nN/\mu m$	Ref. [1]
E_{ECM}	ECM stiffness	Spring	$k_{ECM} = 4500\, nN/\mu m$	Ref. [3]
			$L0_{ECM-pouch} = -0.06\ \mu m$	
			$L0_{ECM-BC} = -0.06\ \mu m$	
			$L0_{ECM-perip} = 0.06\ \mu m$	
E_{vol}	Conserving volume of cytoplasm	Lagrange multiplier	$k_{vol} = 30\, nN/\mu m^2$	Ref. [4]
			$\Omega_{0pouch} = \Omega_{0perip} = 65\ \mu m^2$	
			$\Omega_{0Bc} = 20\ \mu m^2$	

SI Table B. Parameters in the wild type simulations before and after dome shape of wing disc is formed. Other parameters are the same as shown in the Table A.

Parameter	Definition	Before dome shape profile formation	After dome shape profile formation
C_{ECM}	Damping coefficient (resistance to move)	$36\, nN/\mu ms$	$36 \times 10^{3}\, nN/\mu ms$
E_{cont}	Basal actomyosin contractility	$k_{cont} = 9\, nN/\mu m$	$k_{cont} = 0\, nN/\mu m$

SI Table C. Parameters of energy functions representing effects of the actomyosin inhibition by using Latrunculin corresponding to Fig. 7B'. Other parameters are the same as in the last column of SI Table B and in SI Table A.

Energy	Definition	Interaction Type	Values
E_{adhA}	Adhesion between columnar & squamous cells	Spring	$k_{adhA} = 0$

SI Table D. Parameters of energy functions represent effects of ECM removal. The parameters correspond Fig. 8B'-D'. Other parameters are the same as in the last column of SI Table B and SI Table A.

Energy	Definition	Interaction Type	Values
E_{adhB}	Integrin	Spring	$k_{adhB} = 0$
E_{ECM}	ECM stiffness	Spring	$k_{ecm} = 0$

SI Table E. Parameters of energy functions represents effects of ECM removal and actomyosin inhibition through ROCK inhibition. The parameters correspond Figure 9B'-D'. Other parameters are the same as in the last column of SI Table B and SI Table A.

Energy	Definition	Interaction Type	Values
E_{adhA}	Adhesion between columnar and squamous cells	Spring	$k_{adhA} = 0$
E_{adhB}	Integrin	Spring	$k_{adhB} = 0$
E_{ECM}	ECM stiffness	Spring	$k_{ecm} = 0$

References:

1. Nematbakhsh A, Sun W, Brodskiy PA, Amiri A, Narciso C, Xu Z, et al. Multi-scale computational study of the mechanical regulation of cell mitotic rounding in epithelia. PLoS Comput Biol. 2017;13(5):e1005533.
2. Sim JY, Moeller J, Hart KC, Ramallo D, Vogel V, Dunn AR, et al. Spatial distribution of cell–cell and cell–ECM adhesions regulates force balance while maintaining E-cadherin molecular tension in cell pairs. Mol Biol Cell. 2015;26(13):2456–2465.

3. Keller A, Lanfranconi F, Aegerter CM. The influence of geometry on the elastic properties of the Drosophila wing disc. Phys Stat Mech Its Appl. 2018;510:208–218.
4. Pivkin IV, Karniadakis GE. Accurate coarse-grained modeling of red blood cells. Phys Rev Lett. 2008;101(11):118105.
